# Polyketides with potential bioactivities from the mangrove-derived fungus *Talaromyces* sp. WHUF0362

**DOI:** 10.1007/s42995-023-00170-5

**Published:** 2023-03-31

**Authors:** Huawei Lv, Haibo Su, Yaxin Xue, Jia Jia, Hongkai Bi, Shoubao Wang, Jinkun Zhang, Mengdi Zhu, Mahmoud Emam, Hong Wang, Kui Hong, Xing-Nuo Li

**Affiliations:** 1 College of Pharmaceutical Science & Key Laboratory of Marine Fishery Resources Exploitment & Utilization of Zhejiang Province, Zhejiang University of Technology, Hangzhou, 310014 China; 2 School of Pharmaceutical Sciences, Wuhan University, Wuhan, 430072 China; 3 Department of Pathogen Biology & Jiangsu Key Laboratory of Pathogen Biology, Nanjing Medical University, Nanjing, 211166 China; 4 Beijing Key Laboratory of Drug Target Research and New Drug Screening, Institute of Materia Medica, Chinese Academy of Medical Sciences, Beijing, 100700 China; 5 Research Center of Analysis and Measurement, Zhejiang University of Technology, Hangzhou, 310014 China; 6 Department of Phytochemistry and Plant Systematics, National Research Centre, Giza, Egypt

**Keywords:** Mangrove-derived fungus, *Talaromyces* sp., Depsidone, Xanthone, Antimicrobial

## Abstract

Metabolites of microorganisms have long been considered as potential sources for drug discovery. In this study, five new depsidone derivatives, talaronins A-E (**1–5**) and three new xanthone derivatives, talaronins F–H (**6–8**), together with 16 known compounds (**9–24**), were isolated from the ethyl acetate extract of the mangrove-derived fungus *Talaromyces* species WHUF0362. The structures were elucidated by analysis of spectroscopic data and chemical methods including alkaline hydrolysis and Mosher’s method. Compounds **1** and **2** each attached a dimethyl acetal group at the aromatic ring. A putative biogenetic relationship of the isolated metabolites was presented and suggested that the depsidones and the xanthones probably had the same biosynthetic precursors such as chrysophanol or rheochrysidin. The antimicrobial activity assay indicated that compounds **5**, **9**, **10**, and **14** showed potent activity against *Helicobacter pylori* with minimum inhibitory concentration (MIC) values in the range of 2.42–36.04 μmol/L. While secalonic acid D (**19**) demonstrated significant antimicrobial activity against four strains of *H. pylori* with MIC values in the range of 0.20 to 1.57 μmol/L. Furthermore, secalonic acid D (**19**) exhibited cytotoxicity against cancer cell lines Bel-7402 and HCT-116 with IC_50_ values of 0.15 and 0.19 μmol/L, respectively. The structure–activity relationship of depsidone derivatives revealed that the presence of the lactone ring and the hydroxyl at C-10 was crucial to the antimicrobial activity against *H. pylori*. The depsidone derivatives are promising leads to inhibit *H. pylori* and provide an avenue for further development of novel antibiotics.

**Supplementary Information:**

The online version contains supplementary material available at 10.1007/s42995-023-00170-5.

## Introduction

Microbial secondary metabolites have received great attention as a potential resource of lead drugs owing to their productive biological activities and massive chemical diversity (Hai et al. 2021; Xu et al. 2022). Due to the special mangrove environment, including high salinity, low oxygen, nutrient limitation, and drought, mangrove-derived fungi have the biosynthetic potential to produce a variety of unique secondary metabolites (Liang et al. 2019; Nathan et al. 2020; Xu et al. 2014; Zhang et al. 2021). The genus *Talaromyces* (Trichocomaceae) is a sexual state of *Penicillium*, and has the potential to produce depsidones (Zhao et al. 2015; Wu et al. 2015). In the viewpoint of ecologies, the occurrence of *Talaromyces* makes these fungi increasingly regarded as a source of interesting bioactive compounds, leading to the discovery of drugs, such as penicillin, compactin, anti-mycotoxins, and miscellaneous antitumor products (Nicoletti and Trincone 2016; Nicoletti et al. 2018).

The depsidones were a series of compounds derived from depsides by a loss of hydrone in an oxidative cyclization and aroused great pharmacological interest as antimicrobial and cytotoxic agents (Hong et al. 2018; Ureña-Vacas et al. 2022; Yilmaz et al. 2004). Some depsidones act as RecA protein inhibitors by increasing bactericidal activity and reducing antibiotic resistance. Furthermore, depsidones have also targeted the protein FabZ of the bacterial system for fatty acid biosynthesis (FAS) (Alam et al. 2016; McGillick et al. 2016). Depsidones can attenuate cell tumor growth by acting as selective inhibitors of Plk1 activity or directly target antiapoptotic Bcl-2 family proteins (Hong et al. 2018; WilliaNms et al. 2011).

**Fig. 1:**
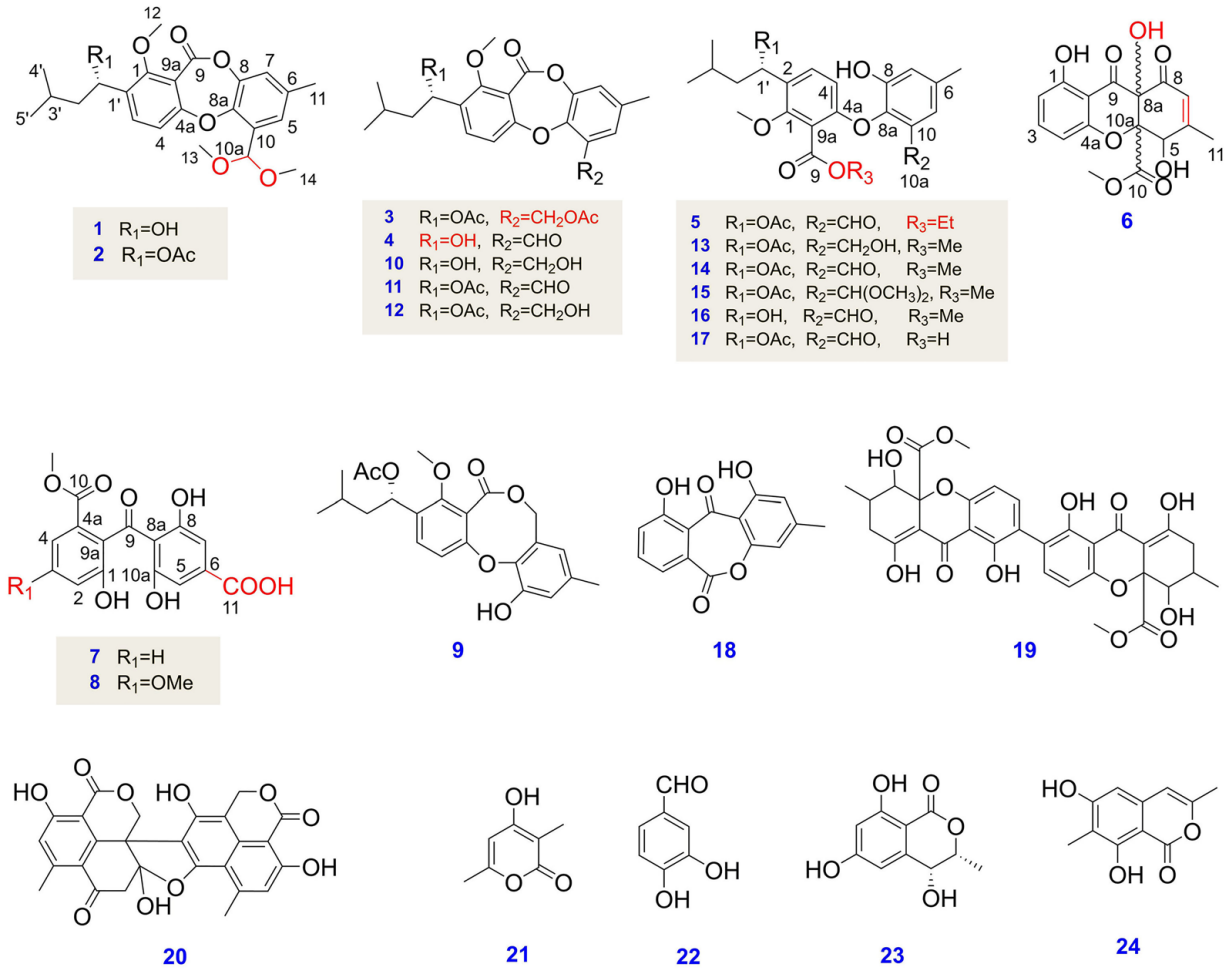
Structures of metabolites isolated from *Talaromyces* sp. WHUF0362

In the current study, the fungus *Talaromyces* sp. WHUF0362, isolated from the mangrove soil sample collected from Yalog Bay, at Sanya, Haian, China, showed potent antimicrobial activities against *Escherichia coli* and *H. pylori* G27. During our search for active secondary metabolites from the marine-derived fungi, the chemical investigation of secondary metabolites of *Talaromyces* sp. WHUF0362 was performed. This work resulted in the purification and identification of five new depsidones, talaronins A-E (**1–5**), and three new xanthone derivatives, talaronins F–H (**6–8**), together with 16 known compounds (**9–24**) (Fig. 1). In addition, the isolated compounds were evaluated for antimicrobial activity (ten Gram-negative bacteria, seven Gram-positive bacteria, a Mycobacterium, and two fungi) and cytotoxic activity (Bel-7402, HCT-116, and A549).

## Results and discussion

The strain *Talaromyces* sp. WHUF0362 was isolated from the mangrove soil sample collected from Yalog Bay, at Sanya, Haian, China. A crude extract of *Talaromyces* sp. WHUF0362 cultivated in the PDA medium exhibited antimicrobial activities against *Escherichia coli* and *H. pylori* G27. A further chemical investigation of the rice fermentation products of *Talaromyces* sp. WHUF0362 was carried out and led to isolation and identification of 24 polyketide derivatives, eight (**1–8**) of which were determined as new by comprehensive analysis of spectroscopic data (1D and 2D NMR, HRESIMS, IR, and UV) and chemical methods including alkaline hydrolysis and Mosher’s method.

**Fig. 2:**
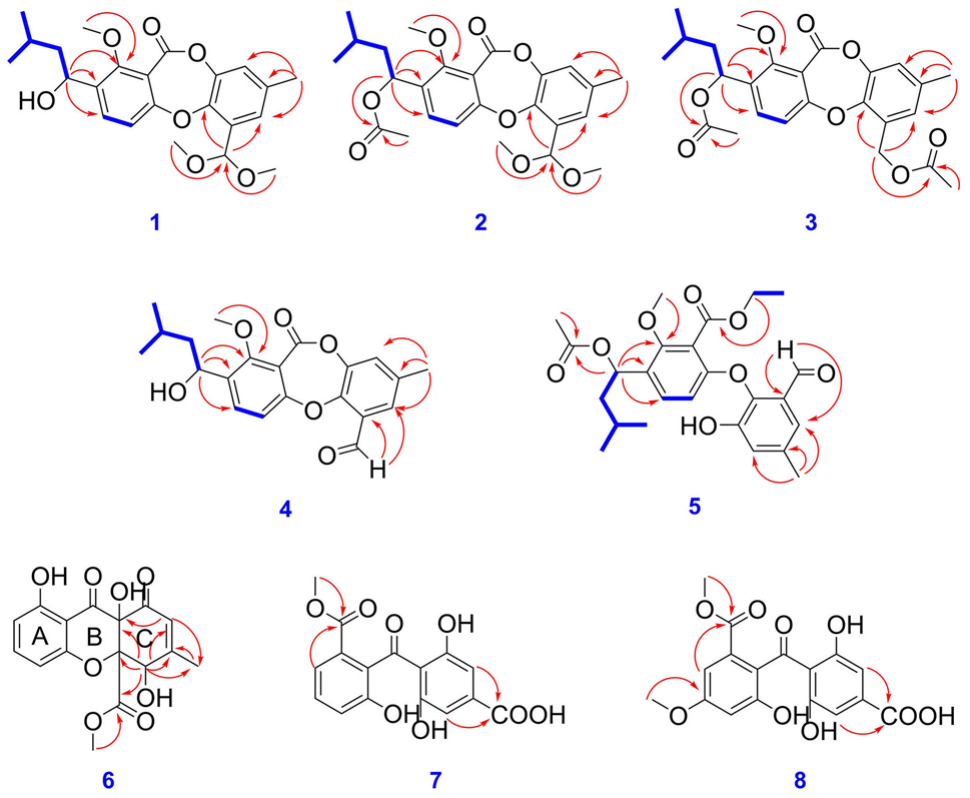
Key HMBC (red arrows) and ^1^H-.^1^H COSY (blue lines) correlations of compounds **1**–**8**

**Fig. 3:**
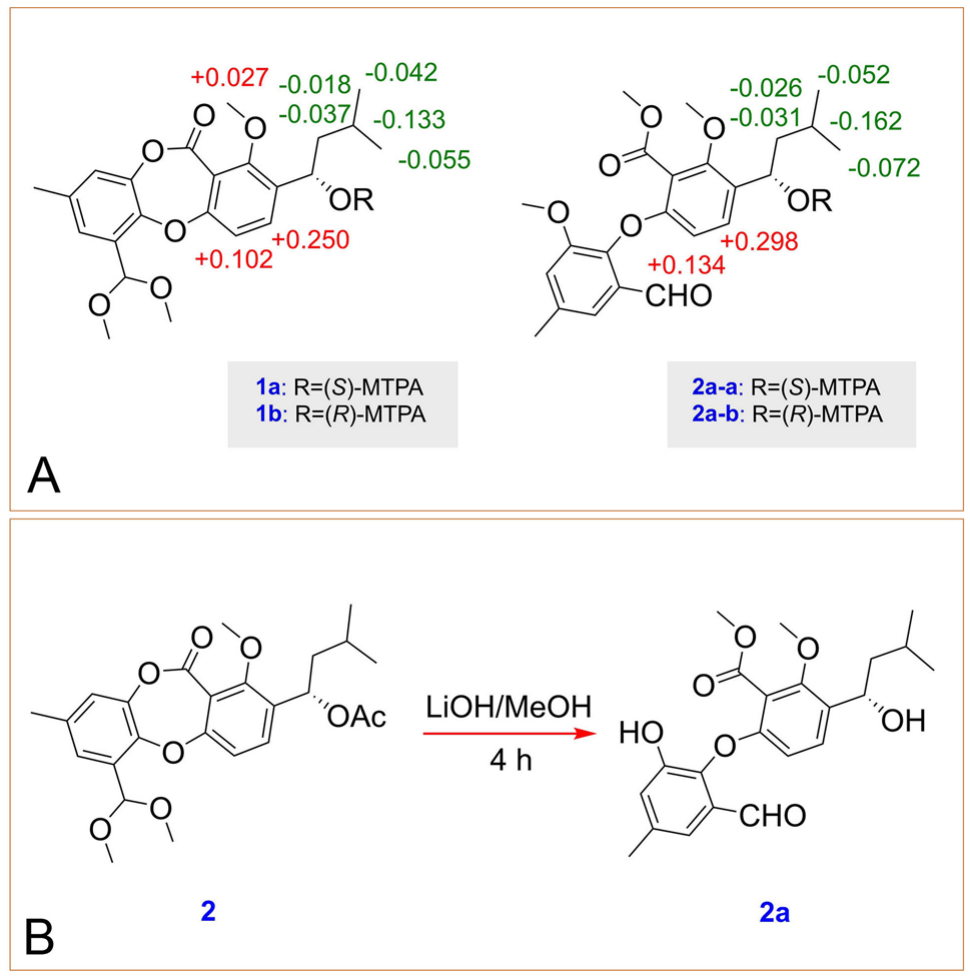
Δ*δ*_(*S*-*R*)_ values for (*S*)- and (*R*)-MTPA esters of compounds **1** and** 2a** (A) and alkaline hydrolysis of compound **2** (B)

Talaronin A (**1**) was obtained as a colorless oil. Its molecular formula was deduced to be C_23_H_28_O_7_ based on high resolution electrospray ionization mass spectroscopy (HRESIMS) data (*m*/*z*: 439.1735 [M + Na]^+^, calcd. for C_23_H_28_O_7_Na: *m*/*z* 439.1727), indicating ten degrees of unsaturation. The ^1^H and ^13^C NMR (Table 1) data indicated that the presence of two aromatic rings [*δ*_H_ 7.56 (d, *J* = 8.6 Hz), 7.19 (d, *J* = 2.1 Hz), 7.08 (d, *J* = 8.6 Hz), 7.04 (d, *J* = 2.1 Hz), *δ*_C_ 115.5, 131.9, 135.8, 158.9, 114.6, 143.9, 121.6, 136.0, 124.8, 130.7, 146.6, and 161.0], three methoxy groups (*δ*_H_ 3.88, 3.37, and 3.35, *δ*_C_ 63.0, 53.6, and 53.5), an ester carbonyl group (*δ*_C_ 161.8), three aliphatic methyls [*δ*_H_ 2.32 (s), 0.97 (d, *J* = 6.5 Hz), 0.94 (d, *J* = 6.7 Hz), *δ*_C_ 21.1, 21.9, and 23.6], three methines [*δ*_H_ 5.81 (s), 5.07 (dd, *J* = 9.2, 4.0 Hz), 1.78 (m),* δ*_C_ 98.8, 67.0, 25.1], and a methylene [*δ*_H_ 1.64 (ddd, *J* = 14.2, 9.2, 5.1 Hz), 1.44 (ddd, *J* = 14.2, 8.8, 4.0 Hz), *δ*_C_ 47.6] by comprehensive analysis of ^1^H and ^13^C nuclear magnetic resonance (NMR) and heteronuclear single quantum coherence (HSQC). The aforementioned ^1^H and ^13^C NMR (Table 1) data which were similar to those of purpactin C′ (**11**) revealed that **1** could be a depsidone derivative (Chen et al. 2015). The main differences were the absence of the signals of an aldehyde and an acetyl in **1**. Instead, it was found to be the presence of two methoxyls and a methine. The heteronuclear multiple bond connectivity (HMBC) correlations from H_3_-13 (*δ*_H_ 3.37) and H_3_-14 (*δ*_H_ 3.35) to C-10a (*δ*_C_ 98.8) confirmed the presence of a dimethyl acetal unit in **1** and the HMBC correlations from H-10a (*δ*_H_ 5.81) to C-5 (*δ*_C_ 124.8) and C-8a (*δ*_C_ 146.6) indicated the dimethyl acetal unit was at C-10a. The ^1^H-^1^H correlation spectroscopy (COSY) displayed one isolated proton spin system for the isoamyl group as shown in Fig. 2. The chemical shift of H-1′ [*δ*_H_ 5.05 (dd, *J* = 9.2, 4.0 Hz)] in **1** implied the presence of a hydroxyl at C-1′ instead of 1′-acetyl group by combined analysis of its HRESIMS data. The configuration of C-1′ was determined on the basis of the Mosher’s method (Zhang et al. 2013). Treatment of **1** with (*R*)- and (*S*)-*α*-methoxy-*α*-(trifluoromethyl) phenylacetyl chloride (MTPA-Cl) gave the (*S*)- and (*R*)-MTPA esters (**1a** and **1b)**, respectively. The ^1^H NMR signals of two MTPA esters were assigned. The absolute configuration was determined to be 1′*S* by the calculation of Δ*δ*_(*S*-*R*)_ values (Fig. 3A). Thus, the structure of **1** was assigned as shown and it was named as talaronin A.

**Table 1:** ^1^H and ^13^C NMR data of compounds **1**, **2**, **3**, and **4** (600 and 150 MHz, *δ* in × 10^–6^)

	**1** (CDCl_3_)		**2** (CDCl_3_)		**3** (MeOH-*d*_*4*_)		**4** (CDCl_3_)	
No	*δ*_H_ (*J*, Hz)	*δ*_C_, type	*δ*_H_ (*J*, Hz)	*δ*_C_, type	*δ*_H_ (*J*, Hz)	*δ*_C_, type	*δ*_H_ (*J*, Hz)	*δ*_C_, type
1		158.9, C		158.9, C		160.2, C		159.3, C
2		135.8, C		132.9, C		134.2, C		136.8, C
3	7.56, d (8.6)	131.9, CH	7.42, d (8.6)	131.6, CH	7.60, d (8.6)	133.1, CH	7.63, d (8.5)	132.6, CH
4	7.08, d (8.6)	115.5, CH	7.05, d (8.6)	115.7, CH	7.17, d (8.6)	116.4, CH	7.05, d (8.5)	114.7, CH
4a		161.0, C		161.1, C		162.6, C		160.6, C
5	7.19, d (2.1)	124.8, CH	7.18, d (2.1)	124.8, CH	7.13, d (2.0)	128.7, CH	7.48, d (2.1)	125.4, CH
6		136.0, C		136.0, C		138.1, C		136.9, C
7	7.04, d (2.1)	121.6, CH	7.03, d (2.2)	121.7, CH	7.15, d (2.0)	122.4, CH	7.32, d (2.2)	127.4, CH
8		143.9, C		143.8, C		145.0, C		144.4, C
8a		146.6, C		146.4, C		148.2, C		151.0, C
9		161.8, C		161.5, C		162.5, C		160.9, C
9a		114.6, C		115.2, C		115.8, C		114.3, C
10		130.7, C		130.6, C		130.3, C		128.6, C
10a	5.81, s	98.8, CH	5.80, s	98.8, CH	5.35, d (12.0) 5.32, d (12.0)	61.7, CH_2_	10.61, s	187.8, CH
11	2.32, s	21.1, CH_3_	2.32, s	21.1, CH_3_	2.36, s	20.8, CH_3_	2.37, s	20.9, CH_3_
12	3.88, s	63.0, CH_3_	3.93, s	63.1, CH_3_	3.92, s	63.4, CH_3_	3.91, s	63.2, CH_3_
13	3.37, s	53.6, CH_3_	3.38, s	53.8, CH_3_				
14	3.35, s	53.5, CH_3_	3.33, s	53.3, CH_3_				
1′	5.05, dd (9.2, 4.0)	67.0, CH	6.12, dd (9.4, 4.3)	68.8, CH	6.14, dd (9.2, 4.7)	70.0, CH	5.07, dd (9.2, 4.0)	66.9, CH
2′	1.64, ddd (14.2, 9.2, 5.1) 1.44, ddd (14.2, 8.8, 4.0)	47.6, CH_2_	1.74, ddd (14.3, 9.4, 5.3) 1.48, ddd (14.1, 8.4, 4.4)	45.2, CH_2_	1.81, ddd (14.3, 9.2, 5.4) 1.52, ddd (14.0, 8.3, 4.7)	46.1, CH_2_	1.63, ddd (14.2, 9.2, 5.1) 1.43, ddd (13.9, 8.9, 4.0)	47.6, CH_2_
3′	1.78, m	25.1, CH	1.62, m	25.0, CH	1.65, m	26.1, CH	1.78, m	25.1, CH
4′	0.94, d (6.7)	23.6, CH_3_	0.92, d (4.1)	23.2, CH_3_	0.97, s	23.4, CH_3_	0.97, d (6.6)	21.9, CH_3_
5′	0.97, d (6.5)	21.9, CH_3_	0.93, d (4.0)	22.0, CH_3_	0.98, s	22.3, CH_3_	0.94, d (6.7)	23.5, CH_3_
1′-OAc			2.04, s	21.3, CH_3_ 170.3, C	2.07, s	20.9, CH_3_ 172.3, C		
10a-OAc					2.10, s	20.8, CH_3_ 172.1, C

Talaronin B (**2**) was obtained as a yellow oil and possessed the molecular formula C_25_H_30_O_8_ determined by HRESIMS ion at *m*/*z* 481.1844 [M + Na]^+^ (calcd. for C_25_H_30_NaO_8_: *m*/*z* 481.1833), indicating a 42 mass unit more than **1**. Comparison of the ^1^H and ^13^C NMR data (Table 1) of **2** with those of **1** revealed that additional acetyl signals (*δ*_H_ 2.04, *δ*_C_ 21.3 and 170.3) appeared in **2**. The downfield shift of H-1′ [*δ*_H_ 6.12 (dd, *J* = 9.4, 4.3 Hz)] in **2** indicated the presence of an acetyl group at C-1′ instead of the 1′-hydroxyl. The HMBC correlation from H-1′ (*δ*_H_ 6.12) to a carbonyl group at *δ*_C_ 170.3 confirmed the above hypothesis (Fig. 2). Compound **2** was hydrolyzed by LiOH in methanol to obtain the hydrolysis product **2a** (Fig. 3B). Compound **2a** was reacted with (*R*)- and (*S*)-MTPA-Cl to yield (*S*)- and (*R*)-MTPA esters (**2a-a** and **2a-b**), respectively. The absolute configuration of **2** was deduced to be 1′*S* by the calculation of Δ*δ*_(*S*-*R*)_ values of the MTPA esters (Fig. 3A). Therefore, compound **2** was determined and named talaronin B.

Talaronin C (**3**) was obtained as a colorless oil with the molecular formula C_25_H_28_O_8_ inferred from its HRESIMS (*m*/*z* 474.2139 [M + NH_4_]^+^, calcd. for C_25_H_32_NO_8_: *m*/*z* 474.2122), accounting for twelve degrees of unsaturation. The general features of the ^1^H and ^13^C NMR spectra resembled those of talaromyone B (**12**) (Cai et al. 2017) except for the presence of an additional acetyl moiety. The significant downfield shift of H-10 and the key correlations from H-10 (*δ*_H_ 5.35, 5.32) to a carbonyl group at *δ*_C_ 172.1 suggested the acetyl group should be located at C-10. Thus, the structure of **3** was determined as shown and given the name talaronin C.

Talaronin D (**4**) was isolated as a yellow oil with the molecular formula of C_21_H_22_O_6_ based on its HRESIMS data (*m*/*z*: 369.1337 [M-H]^–^, calcd. for C_21_H_22_O_6_: *m*/*z* 369.1344), accounting for eleven degrees of unsaturation. Analyses of the ^1^H and ^13^C NMR (Table 1) signals of **4** indicated the presence of an isoamyl (*δ*_H_ 5.07, 1.63, 1.43, 1.78, 0.97, 0.94; *δ*_C_ 66.9, 47.6, 25.1, 21.9, 23.5), an aldehyde group (*δ*_H_ 10.61, *δ*_C_ 187.8), and a methoxyl (*δ*_H_ 3.91, *δ*_C_ 63.2). The 1D NMR spectroscopic data of **4** were similar to those obtained from **1** beyond the absence of a dimethyl acetal unit and the presence of an aldehyde group in **4**. The aldehyde group was determined to be at C-10 as evident by HMBC correlations from H-10 (*δ*_H_ 10.61) to C-5 (*δ*_C_ 125.4) and C-10 (*δ*_C_ 128.6). Unambiguously, the structure of **4** was assigned as shown and named to be talaronin D.

Talaronin E (**5**) was isolated as a colorless oil and assigned a molecular formula of C_25_H_30_O_8_ based on the HRESIMS data (*m*/*z*: 457.1879 [M-H]^–^, calcd. for C_25_H_29_O_8_: *m*/*z* 457.1868), implying eleven degrees of unsaturation. Analyses of the ^1^H NMR data with the aid of HSQC and ^13^C NMR spectra revealed the existence of an aldehyde group (*δ*_H_ 10.20, *δ*_C_ 189.5), two aromatic rings [*δ*_H_ 7.27 (d, *J* = 8.8 Hz), 7.26 (d, *J* = 2.1 Hz), 7.10 (d, *J* = 2.1 Hz), 6.49 (d, *J* = 8.7 Hz)], an ester group (*δ*_C_ 167.5) and a acetyl (*δ*_H_ 2.03, *δ*_C_ 21.3, *δ*_C_ 170.4). These groups were attributed to the eleven degrees of unsaturation given by HRESIMS, which indicated that there was no lactonic ring in **5** compared to the depsidone. 1D NMR spectra of **5** showed a close similarity with those of secopenicillide B (**14**) (Komai et al. 2006) with exceptions of the absence of the methoxyl group and the presence of an ethoxyl [*δ*_H_ 4.53 (q, *J* = 7.1 Hz) 1.47 (t, *J* = 7.2 Hz)]. The HMBC correlation from *δ*_H_ 4.53 to C-9 (*δ*_C_ 167.5) suggested the ethoxyl moiety was bonded to the C-9 carbonyl. Therefore, compound **5** was named as talaronin E.

Talaronin F (**6**) was isolated as a brown oil with a molecular formula of C_16_H_14_O_8_ revealed by analysis of its HRESIMS (*m*/*z* 333.0635 [M-H]^–^, calcd. for C_16_H_13_O_8_: *m*/*z* 333.0616), implying ten degrees of unsaturation in **6**. Comparison of the ^1^H NMR and ^13^C NMR data of **6** with those of ergochrome E (Yong et al. 2021) hinted **6** should be an xanthone analogue of ergochrome E. The 1D NMR data (Table 2) were fully assigned by detailed analysis of the HSQC and HMBC spectra. The presence of three aromatic protons [*δ*_H_ 7.50 (t, *J* = 8.3 Hz), 6.63 (d, *J* = 8.2 Hz), 6.61 (d, *J* = 8.3 Hz)] indicated an AMX-spin system in **6**. The HMBC correlations from H_3_-11 (*δ*_H_ 2.17) and H-5 (*δ*_H_ 4.65) to C-6, 7 (*δ*_C_ 156.5, 128.4) denoted the aliphatic carbons of C-5 and C-6 in ergochrome E were replaced by two olefinic carbons in **6**. The chemical shift of *δ*_C_ 191.2 in **6** implies an *α*, *β*-unsaturated ketone by analysis of the chemical shift of C-6 and C-7 combined with the weak HMBC correlation of H_3_-11/C-8. The HMBC correlation from H-7 to C-8a signified a hydroxyl at C-8a. Thus, compound **6** was named talaronin F.

**Table 2:** ^1^H and ^13^C NMR data of compounds **5**, **6**, **7**, and **8** (600 and 150 MHz, *δ* in × 10^–6^)

	**5** (CDCl_3_)		**6** (MeOH-*d*_*4*_)		**7** (MeOH-*d*_*4*_)		**8** (MeOH-*d*_*4*_)	
No	*δ*_H_ (*J*, Hz)	*δ*_C_, type	*δ*_H_ (*J*, Hz)	*δ*_C_, type	*δ*_H_ (*J*, Hz)	*δ*_C_, type	*δ*_H_ (*J*, Hz)	*δ*_C_, type
1		155.6, C		159.0, C		154.9, C		157.3, C
2		131.4, C	6.61, d (8.3)	112.0, CH	7.06, dd (7.8, 0.9)	121.0, CH	6.63, d (2.4)	106.3, CH
3	7.27, d (8.8)	129.9, CH	7.50, t (8.3)	139.5, CH	7.30, t (8.0)	129.9, CH		162.2, C
3-Me							3.85, s	56.0, CH_3_
4	6.49, d (8.7)	112.0, CH	6.63, d (8.2)	108.6, CH	7.50, dd (7.8, 0.9)	121.6, CH	6.98, d (1.8)	107.0, CH
4a		155.8, C		164.2, C		129.3, C		131.5, C
5	7.26, d (2.1)	119.7, CH	4.65, s	72.1, CH	6.90, s	108.7, CH	6.91,s	108.9, C
6		137.4, C		156.5, C		u.b		u.b
7	7.10, d (2.1)	124.9, CH	6.03, d (1.2)	128.4, CH	6.90, s	108.7, CH	6.91,s	108.9, C
8		149.8, C		191.2, C		162.9, C		162.4, C
8a		142.6, C		70.7, C		113.7, C		115.1, C
9		167.5, C		192.8, C		203.0, C		202.5, C
9-OEt	4.53, q (7.1) 1.47, t (7.2)	63.0, CH_2_ 14.3, CH_3_						
9a		118.4, C		107.6, C		134.4, C		126.1, C
10	10.20, s	189.5, CH		170.5, C		168.1, C		168.3, C
10a		130.2, C		85.0, C				
11	2.35, s	21.2, CH_3_	2.17, d (1.2)	21.8, CH_3_		175.5,C		171.3, C
12	3.97, s	63.1, CH_3_	3.65, s	54.2, CH_3_	3.72, s	52.4, CH_3_	3.66, s	52.6, CH_3_
1’	6.10, dd (9.4, 4.5)	68.6, CH						
1′-OAc	2.03, s	170.4, C 21.3, CH_3_						
2’	1.73, ddd (14.3, 9.3, 5.3) 1.47, m	45.6, CH_2_						
3′	1.62, m	25.0, CH						
4′	0.94, d (4.0)	22.0, CH_3_						
5′	0.93, d (4.1)	23.2, CH_3_						

*u.b.* unobserved

Talaronin G (**7**) was obtained as a yellow solid. The molecular formula was determined to be C_16_H_12_O_8_ based on an ion at *m*/*z* 331.0458 [M-H]^–^ by the HRESIMS, indicating eleven degrees of unsaturation. The ^1^H NMR (Table 2) spectrum exhibited an AMX-spin system [*δ*_H_ 7.50 (dd, *J* = 7.8, 0.9 Hz), 7.30 (t, *J* = 8.0 Hz), 7.06 (dd, *J* = 7.8, 0.9 Hz)], two overlapping aromatic proton signals (*δ*_H_ 6.90), and a methoxyl (*δ*_H_ 3.72). The ^13^C NMR data revealed the presence of three carboxyl groups, two aromatic rings, and a methoxyl by taking the ^1^H NMR and HSQC spectroscopic data into account. The general features of the ^1^H and ^13^C NMR spectra of **7** were similar to those of the known compound methyl peniphenone (Liu et al. 2016). The main difference was that there were two overlapping aromatic proton signals in **7** instead of the AMX-spin system, which was in accordance with a symmetrically substituted aromatic ring. The HMBC correlation from H-5, 7 (*δ*_H_ 6.90) to a carbonyl resonance (*δ*_C_ 175.5) suggested the carboxyl was located at C-6 combined with the analysis of HRESIMS data. Hence, the structure of **7** was established as shown and assigned the name talaronin G.

Talaronin H (**8**), a yellow solid, was found to possess a molecular formula of C_17_H_14_O_9_ from HRESIMS ion at *m*/*z* 361.0547 [M-H]^–^ (Calcd. for C_17_H_13_O_9_, 361.0565). The ^1^H and ^13^C NMR data (Table 2) suggested the presence of a characteristic ketone carboxyl, two aromatic rings, and two methoxy groups. 1D NMR spectrum of **8** was also in accordance with **7** except for an additional methoxyl and the absence of the AMX-spin system. The HMBC correlations between H-3-Me (*δ*_H_ 3.85) and C-3 (*δ*_C_ 162.2), H-4 (*δ*_H_ 6.98)/H-2 (*δ*_H_ 6.63) and C-3 (*δ*_C_ 162.2) indicated the methoxy group was placed at C-3. The structure of **8** was identified as shown and named talaronin H.

The known compounds were identified as purpactin A (**9**) (Sy-Cordero et al. 2015), talaromyone A (**10**) (Cai et al. 2017), purpactin C′ (**11**) (Chen et al. 2015), talaromyone B (**12**) (Cai et al. 2017), secopenicillide A (**13**) (Komai et al. 2006), secopenicillide B (**14**) (Komai et al. 2006), talaromycin C (**15**) (Chen et al. 2015), deacetyl talaromycin C (**16**) (Wu et al. 2016), tenellic acid C (**17**) (Cai et al. 2017), alternaphenol B (**18**) (Shen et al. 2017), secalonic acid D (**19**) (Hong 2011), bacillisporin C (**20**) (Dramae et al. 2020), 4-hydroxy-3,6-dimethyl-2-pyrone (**21**) (Smetanina et al. 2017), 3,4-dihydroxybenzaldehyde (**22**), 4,6-dihydroxymellein (**23**) (Takenaka et al. 2011), and similanpyrone B (**24**) (Prompanya et al. 2014) based on their NMR and MS data as well as comparison of their spectroscopic data with those published before.

Compared with the reported depsidone derivatives, the main differences of the five new depsidone derivatives are in the different substituents of C-10, C-9, and C-1′. The known depsidones usually have an aldehyde group at C-10. However, compounds **1** and **2** each contain a dimethyl acetal group at C-10 and the aldehyde group in compound **3** was reduced to a hydroxymethyl group. This provided clues for us to speculate on the biosynthetic pathway of the depsidone. The skeleton of depsidones was produced from acetyl- and malonyl-coenzyme A by nonreducing polyketide synthase (PKS) (Cox 2007; Xu et al. 2014). It was accepted that the depsidones involved the oxidative coupling of benzophenone to give spirobenzofuran-1,2′-cyclohexa-3′,5′-diene-2′,3-dione as an intermediate, which in turn rearranged to the depsidone (Nishida et al. 1992; Xu et al. 2014). Most of the isolates herein we obtained could be divided into two series, the depsidone derivatives (**1**–**5** and **9**–**18**) and the xanthone derivatives (**6–8** and **19**). Both groups probably had the same biosynthetic precursors such as chrysophanol or rheochrysidin. The oxidation product of chrysophanol/rheochrysidin was methylated to offer compounds **6** and **7** (Wei and Matsuda 2020). Cyclization of the oxidation product gave compounds **6** and **18** (Frisvad et al. 2020) or under the action of dimerase to obtain compound **19** (Wei et al. 2021). In addition, the intermediate of spirane was obtained by PKS (Xu et al. 2014), and the rearrangement occurred due to the instability of the spiroane structure (Nishida et al. 1991). The isoprenylation of the rearrangement product was then methylated and/or acetylated to give compounds **1**–**3** and **10**–**12** or directly methylated and/or acetylated to yield compounds **5** and **13**–**17** (Kikuchi et al. 2012; Masters and Bräse 2012). The proposed biogenetic relationship of the isolated metabolites was shown in Supplementary Fig. S68.

The biosynthetic pathways of **3**–**5** were expected to be the same as that of **1**. Thus, the absolute configuration of C-1′ in **3**–**5** was proposed to be *S*.

All of the isolates with adequate amount were evaluated for their antimicrobial activities and cytotoxicity activities (Supplementary Table S1, Table S2, and Table S3). Compounds **5**, **9**, **10**, and **14** showed antibacterial activity against *H. pylori* with MIC values in the range from 2.42 to 36.04 μmol/L, with amoxicillin as positive control with MIC values of 0.14 to 38.14 μmol/L. Compound **11** showed antibacterial activity against *Staphylococcus aureus* NEWMAN with an MIC value of 38.83 μmol/L (Table 3). Particularly, compound **19** showed significant antimicrobial activity against *H. pylori* with MIC values of 0.20 to 1.57 μmol/L. In addition, compound **19** preeminently inhibited cancer cell lines Bel-7402 and HCT-116 with IC_50_ values of 0.15 and 0.19 μmol/L compared with 5-fluorouracil as a positive control with IC_50_ values of 13.69 and 12.23 μmol/L, respectively. According to their structural characteristics, the isolated depsidone derivatives could be divided into two categories: with lactone ring and without lactone ring. Interestingly, **5** and **13**–**17** without lactone ring exhibited the weak anti-*H. pylori* activity with MIC values higher than 32.65 μmol/L. While **9** and **10** possessed the lactone ring showed anti-*H. pylori* activity with MIC values of 2.41 to 10.75 μmol/L. It was suggested that the presence of the lactone ring in depsidone derivatives was related to the anti-*H. pylori* activity. Furthermore, **4** displayed no activity against *H. pylori*; whereas **10**, reduzate of **4,** had better anti-*H. pylori* activity with MIC values of 5.38 to 10.75 μmol/L. Additionally, the substituted group at C-10 in **9** was a hydroxyl which made it exhibit better anti-*H. pylori* activity with MIC values of 2.41 to 4.83 μmol/L. The above evidence indicated that the presence of the lactone ring and the hydroxyl at C-10 played an important role for antimicrobial activity against *H. pylori*. A previous study (Cai et al. 2017) indicated talaromyone B (**12**) possessed inhibitory activity against *Bacillus subtilis*. This work first presented the inhibitory activity against *H. pylori* of the depsidone analogues and provided an avenue for the further development of novel antibiotics.

**Table 3:** The results of antimicrobial activities (MIC value, μmol/L)

Compounds^a^	*H. pylori* 26,695	*H. pylori* G27	*H. pylori* 159	*H. pylori* 129	*S. aureus* NEWMAN
5	34.93	34.93	–	–	–
9	–	4.83	–	2.42	–
10	10.75	10.75	5.38	5.38	–
11	–	–	–	–	38.83
14	36.04	36.04	–	–	–
19	0.20	0.20	1.57	1.57	–

^a^The compounds that did not show activity at maximum concentrations were not shown in table

## Conclusions

In summary, five new depsidones, talaronins A-E (**1–5**), and three new xanthone derivatives, talaronins F–H (**6–8**), together with 16 known compounds were isolated from the culture of the mangrove-derived fungus *Talaromyces* sp. WHUF0362. Most of the isolates, could be divided into two series of compounds, the depsidone derivatives (**1–5** and **9–17**) and the xanthone derivatives (**6–8**, **18** and **19**), and all of them probably had the same biosynthetic precursors, chrysophanol or rheochrysidin. In the bioactivity assays, secalonic acid D (**19**) demonstrated promising inhibitory activity against the cancer cell lines Bel-7402 and HCT-116 with IC_50_ 0.15 and 0.19 μmol/L, respectively. In additional, secalonic acid D (**19**) showed significant antimicrobial activity against four strains of *H. pylori* with MIC values of 0.20 to 1.57 μmol/L. In addition, the investigated isolates **5**, **9**, **10**, and **14** showed potential activity against *H. pylori* with MIC values of 2.42 to 36.04 μmol/L. The structure–activity relationship of depsidones revealed that the presence of the lactone ring and the hydroxyl at C-10 was crucial to the antimicrobial activity against *H. pylori*. These promising biological findings could provide an optimistic direction for finding new drugs against *H. pylori*.

## Materials and methods

### General Experimental Procedure

All the 1D and 2D NMR spectra were obtained by a Bruker AVANCE III 600 MHZ spectrometer with TMS as an internal standard (Bruker company, Switzerland). The HRESIMS data were obtained on an Agilent 6210 TOF MS system (Agilent Technologies, Santa Clara, CA, USA) or AB SCIEX Triple TOF 5600^+^ (AB SCIEX, USA). Optical rotations were measured by a JASCO P-1020 polarimeter (Jasco Tokyo Japan). UV spectra were performed in MeOH by using a Shimadzu UV spectrometer-1800 (Shimadzu Corp., Kyoto, Japan). IR spectra (KBr) were obtained on a Nicolet 6700 FT-IR spectrometer (Thermo Electric Nicoli, United States). Semipreparative high performance liquid chromatography (HPLC) was performed by an Agilent 1260 separation system with an Aglient ZORBAC SB-C_18_ column (5 μm, 250 mm × 9.4 mm, 3 mL/min). Sephadex LH-20 gel (GE Healthcare, Uppsala, Sweden) and MCI gel (Mitsubishi Chemical Corp., Japan) were used in column chromatography. And silica gel (200–300 mesh for column chromatography, GF254 for TLC) was supplied by the Yantai Zhifu Huanwu Silicone Factory, Yantai, China.

### Fungal material

The fungal strain *Talaromyces* sp. WHUF0362 was isolated from a mangrove soil sample collected from Yalog Bay, at Sanya, Haian, China, in Dec, 2018. The strain was selected by strong and selective activity against microbial pathogens during assays against *E. coli* CCTCC AB 93,154, *S. aureus* CCTCC AB 91,093 and *Candida albus* CCTCC AY 206,001, and presented serious peaks at UV absorption of 200 nm, 254 nm, and 380 nm. The fungus was identified as *Talaromyces* sp. according to its morphological characteristics and ITS gene sequences (NCBI accession: NR_147424.1). A reference culture of *Talaromyces* sp. WHUF0362 maintained at − 80 °C is stored in Wuhan University, China.

### Fermentation

The fungi were cultured in liquid medium (soluble starch 15 g, glucose 5 g, peptone 5 g, yeast extract 5 g, (NH_4_)_2_SO_4_ 0.5 g, K_2_HPO_4_ 0.5 g, NaCl 0.5 g, MgSO_4_ 0.5 g, CaCO_3_ 1 g, water 1 L, pH 7.5) as a seed solution, and then 5 mL seed solution was inoculated into 60 × 1000 mL glass culture flasks, each containing solid rice medium (rice 80 g, distilled water 120 mL). The fungi were statically fermented for 15 days in room temperature.

### Extraction and Isolation

Fermentation products were extracted three times with EtOAc by soaking overnight. The crude extraction (83.77 g) was obtained by vacuum distillation. This extract was fractionated by silica gel column chromatography using the PE (petroleum ether, 60–90 °C) and the EtOAc gradient system (1:0 to 0:1, *v*/*v*) to give 7 fractions (A–G). Fraction D was applied to the silica gel column chromatography eluting with a step gradient of petroleum ether (PE):EtOAc (10:1 to 0:1, *v*/*v*) to obtain 9 fractions (D1–D9). Fraction D6 was purified by semipreparative HPLC (MeOH-H_2_O, 45:55, *v*/*v*) to yield **7** (5.8 mg) and **22** (2.1 mg). Fraction D2 was submitted to silica gel column chromatography eluting with PE:EtOAc (15:1 to 1:1, *v*/*v*) and purified by HPLC (MeOH-H_2_O, 68:32, *v*/*v*) to afford **9** (25.6 mg), **13** (5.8 mg), **19** (33.7 mg) and a mixture which was further purified by semipreparative HPLC (MeOH-H_2_O, 35:65, *v*/*v*) to afford **6** (10.5 mg). Fraction D3 was fractionated further by the silica gel column chromatography into two main subfractions (D3a and D3b) eluting with the gradient CH_2_Cl_2_:MeOH (40:1 to 5:1, *v*/*v*). Fraction D3a was purified by semipreparative HPLC (MeOH-H_2_O, 55:45, *v*/*v*) to yield **10** (8.5 mg). Fraction D3b was applied to silica gel column chromatography eluting with CH_2_Cl_2_:MeOH (70:1 to 20:1, *v*/*v*) and further purified by semipreparative HPLC (MeOH-H_2_O, 25:75, *v*/*v*) to yield **21** (2.6 mg). Fraction D4 was passed over a Sephadex LH-20 column, which was eluted with MeOH, and further purified by semipreparative HPLC (MeOH-H_2_O, 30:70, *v*/*v*) to give **23** (3.1 mg). Fraction D5 was applied to a Sephadex LH-20 column eluting with MeOH and further purified by semipreparative HPLC (MeOH-H_2_O, 37:63, *v*/*v*) to afford **8** (5.9 mg). Fraction C was separated into ten fractions (C1 to C10) by column chromatography on MCI gel eluting with a step gradient of MeOH-H_2_O (20:80 to 100:0, *v*/*v*). Fraction C10 was applied to a silica gel column eluting with PE:EtOAc (10:1 to 5:1, *v*/*v*) and further purified by semipreparative HPLC (MeOH-H_2_O, 80:20, *v*/*v*) to yield **2** (13.4 mg) and **11** (14.0 mg). Fraction C10-2 was applied to the semipreparative HPLC (MeOH-H_2_O, 80:20, *v*/*v*) to afford **3** (4.0 mg). Fraction C9 was fractionated by silica gel column chromatography eluting with PE:EtOAc (10:1 to 5:1, *v*/*v*) to give eight subfractions (C9-1 to C9-8). Compound **5** (9.8 mg) was obtained by semipreparative HPLC (MeOH-H_2_O, 69:31, *v*/*v*) from fraction C9-3. Fraction C9-4 was submitted to Sephadex LH-20 column eluting with MeOH and further purified by semipreparative HPLC (CH_3_CN-H_2_O, 65:35, *v*/*v*) to yield **15** (13.0 mg). Fraction C9-5 was applied to semipreparative HPLC (MeOH-H_2_O, 71:29, *v*/*v*) to afford **1** (7.0 mg). A precipitate from fraction C9-7 was washed with chloroform to give **20** (200.0 mg). Fraction C8 was applied to the silica gel column eluting with PE:EtOAc (15:1 to 3:1, *v*/*v*) to give **14** (3.0 g) and fraction C8-1. Fraction C8-1 was performed on the Sephadex LH-20 column eluting with MeOH and subsequently purified by semipreparative HPLC (MeOH-H_2_O, 65:35, *v*/*v*) to yield **4** (5.6 mg), **12** (4.4 mg) and **16** (6.9 mg). Fraction C7 was applied to a silica gel column eluting with a gradient of PE:EtOAc (5:1 to 3:1, *v*/*v*) to obtain **17** (933.6 mg) and a mixture containing **24** (2.0 mg) which was further isolated by semipreparative HPLC (MeOH-H_2_O, 72:28, *v*/*v*). Fraction C3 was separated by semipreparative HPLC (MeOH-H_2_O-formic acid, 70:30:0.1, *v*/*v*) to give **18** (7.9 mg).

Talaronin A (**1**): colorless oil: [*α*]20 D – 22.2 (*c* 0.14, CHCl_3_); UV (MeOH) *λ*_max_ (log *ε*) 210 (4.38), 279 (3.41) nm; IR (KBr) *v*_max_ 3445, 2955, 2932, 1748, 1596, 1470, 1275, 1053 cm^−1^; HRESIMS *m*/*z* 439.1735 [M + Na]^+^ (calcd. for C_23_H_28_O_7_Na,439.1727); ^1^H NMR and ^13^C NMR data in Table 1.

Talaronin B (**2**): yellow oil; [*α*]20 D + 8.8 (*c* 0.11, CHCl_3_); UV (MeOH) *λ*_max_ (log *ε*) 210 (4.44), 299 (3.38) nm; IR (KBr) *v*_max_ 2957, 2917, 2849, 1747, 1471, 1279, 1234, 1050 cm^−1^; HRESIMS *m*/*z* 481.1844 [M + Na]^+^ (calcd. for C_25_H_30_O_8_Na, 481.1833); ^1^H NMR and ^13^C NMR data in Table 1.

Talaronin C (**3**): colorless oil: [*α*]20 D − 8.8 (*c* 0.07, CH_3_OH); UV (MeOH) *λ*_max_ (log *ε*) 210 (4.53), 280 (3.55) nm; IR (KBr) *v*_max_ 2958, 1743, 1597, 1471, 1235, 1050 cm^−1^; HRESIMS *m*/*z* 474.2139 [M + NH_4_]^+^ (calcd. for C_25_H_32_NO_8_, 474.2122); ^1^H NMR and ^13^C NMR data in Table 1.

Talaronin D (**4**): yellow oil; [*α*]20 D − 20.4 (*c* 0.11, CHCl_3_); UV (MeOH) *λ*_max_ (log *ε*) 208 (4.27), 278 (3.29) nm; IR (KBr) *v*_max_ 3445, 2955, 2919, 1697, 1747, 1595, 1469, 1307, 1276, 1050 cm^−1^; HRESIMS *m*/*z* 369.1337 [M-H]^−^ (calcd. for C_21_H_21_O_6_, 369.1344); ^1^H NMR and ^13^C NMR data in Table 1.

Talaronin E (**5**): colorless oil: [*α*]20 D − 30.4 (*c* 0.17, CHCl_3_); UV (MeOH) *λ*_max_ (log *ε*) 210 (4.56), 268 (3.84) nm; IR (KBr) *v*_max_ 2957, 1738, 1694, 1475, 1279, 1236, 1049 cm^−1^; HRESIMS *m*/*z* 457.1879 [M-H]^–^ (calcd. for C_25_H_29_O_8_, 457.1868); ^1^H NMR and ^13^C NMR data in Table 2.

Talaronin F (**6**): brown oil; [*α*]20 D + 62.7 (*c* 0.10, CH_3_OH); UV (MeOH) *λ*_max_ (log *ε*) 210 (3.99), 276 (3.55), 354 (3.13) nm; IR (KBr) *v*_max_ 3390, 2918, 2849, 1730, 1698, 1626, 1581, 1461, 1383,1030,816 cm^−1^; HRESIMS *m*/*z* 333.0635 [M-H]^–^ (calcd. for C_16_H_13_O_8_, 333.0616), ^1^H NMR and ^13^C NMR data in Table 2.

Talaronin G (**7**): yellow solid; UV (MeOH) *λ*_max_ (log *ε*) 234 (3.78), 288 (3.83) nm; IR (KBr) *v*_max_ 3390, 2922, 1722, 1600, 1558, 1488, 1405, 1385, 1292, 1197, 1015, 790, 761 cm^−1^; HRESIMS *m*/*z* 331.0459 [M-H]^–^ (calcd. for C_16_H_11_O_8_, 331.0448); ^1^H NMR and ^13^C NMR data in Table 2.

Talaronin H (**8**): yellow solid; UV (MeOH) *λ*_max_ (log *ε*) 231 (3.82), 289 (3.86) nm; IR (KBr) *v*_max_ 3424.79, 2919, 2850, 1721, 1600, 1558, 1489, 1385, 1293, 1197, 1079, 1015, 790, 761 cm^−1^; HRESIMS *m*/*z* 361.0547 [M-H]^–^ (calcd. for C_17_H_13_O_9_, 361.0565); ^1^H NMR and ^13^C NMR data in Table 2.

### Antimicrobial assays

Ten Gram-negative bacteria: *Salmonella typhimurium* 14028 s, *Shigella dysenteriae*, *H. pylori* 26,695, *H. pylori* G27, *H. pylori* 159, *H. pylori* 129, *E. coli* MG1655, *Pseudomonas aeruginosa* PAO1, *Acinetobacter baumannii* (ATCC 19,606), and *Klebsiella neumoniae* (ATCC 35,657); seven Gram-positive bacteria: *S. aureus* (ATCC 25,923), *S. aureus* NEWMAN, *S. aureus* USA300, *S. aureus* NRS 271, *Enterococcus faecalis* FA2-2, *Enterococcus Faecium* (ATCC 19,434), and *Bacillus subtilis* 168; two fungi: *Candidia albicans* (ATCC SC5314) and *Candidia albicans clinical isolates* YY-1–4, and a Mycobacterium: *Mycolicibacterium smegmatis* (ATCC 607) were used for the antimicrobial assay. The antimicrobial assay and the determination of the MIC values were performed following the broth microdilution method in 96-well plates which was an established protocol (Lv et al. 2021). A detailed protocol can be found in the supporting information.

### Cytotoxicity assay

Compounds were tested for cytotoxicity against human lung epithelial carcinoma (A-549), human hepatocellular carcinoma (Bel-7402), and human colon cancer (HCT-116) cell lines by using the SRB assay. All of the cancer cell lines were purchased from American Type Culture Collection (ATCC). The inhibitory rates of cell proliferation (%) were calculated as [1-(A_treated_/A_control_)] × 100%, and three independent repeated trials were conducted for each compound (*n* = 3). The IC_50_ values were determined with the Logit method from the results of six concentrations of each compound (Wu et al. 2021). A detailed protocol can be found in the supporting information.

## Supplementary Information

Below is the link to the electronic supplementary material.

Supplementary file1 (DOC 5625 KB)

## Data Availability

The data that supports the findings of this study are included in this published article (and its supplementary information files).
