# Supplementary material for: Polyketides with potential bioactivities from the mangrove-derived fungus Talaromyces sp. WHUF0362
Source: Mar Life Sci Technol. 2023 Mar 31;5(2):232–41. doi: 10.1007/s42995-023-00170-5 (PMC10232383; doi:10.1007/s42995-023-00170-5)
Supplement: Supplementary file 1 — Supplementary file1 (DOC 5625 KB) [file 42995_2023_170_MOESM1_ESM.doc]

**Supporting Materials**

Polyketides with potential bioactivities from the mangrove-derived fungus *Talaromyces* sp. WHUF0362

Huawei Lv^1^, Haibo Su^1^, Yaxin Xue^2^, Jia Jia^3^, Hongkai Bi^3^, Shoubao Wang^4^, Jinkun Zhang^1^, Mengdi Zhu^5^, Mahmoud Emam^1,6^, Hong Wang*^1^, Kui Hong*^2^, and Xing-Nuo Li*^1^

^1^ College of Pharmaceutical Science & Key Laboratory of Marine Fishery Resources Exploitment & Utilization of Zhejiang Province, Zhejiang University of Technology, Hangzhou 310014, China

^2^ School of Pharmaceutical Sciences, Wuhan University, Wuhan 430072, China

^3^ Department of Pathogen Biology & Jiangsu Key Laboratory of Pathogen Biology, Nanjing Medical University, Nanjing 211166, China

^4^ Beijing Key Laboratory of Drug Target Research and New Drug Screening, Institute of Materia Medica, Chinese Academy of Medical Sciences, Beijing 100700, China

^5^ Research Center of Analysis and Measurement, Zhejiang University of Technology, Hangzhou 310014, China

^6^ Department of Phytochemistry and Plant Systematics, National Research Centre, Giza, Egypt

**List of Supporting Material**

[**Fig. S1** HRESIMS spectrum of **1**. 5](#__RefHeading___Toc124370366)

[**Fig. S2** ^1^H NMR (600 MHz) spectrum of **1** in CDCl_3_**.** 5](#__RefHeading___Toc124370367)

[**Fig. S3** ^13^C NMR (150 MHz) spectrum of **1** in CDCl_3_**.** 6](#__RefHeading___Toc124370368)

[**Fig. S4** HSQC (600 MHz) spectrum of **1** in CDCl_3_. 6](#__RefHeading___Toc124370369)

[**Fig. S5** HMBC (600 MHz) spectrum of **1** in CDCl_3_. 7](#__RefHeading___Toc124370370)

[**Fig. S6** ^1^H-^1^H COSY (600 MHz) spectrum of **1** in CDCl_3_. 7](#__RefHeading___Toc124370371)

[**Fig. S7** UV spectrum of **1** in MeOH. 8](#__RefHeading___Toc124370372)

[**Fig. S8** IR spectrum of **1**. 8](#__RefHeading___Toc124370373)

[**Fig. S9** ^1^H NMR (600 MHz) spectrum of (*S*)-MTPA ester of **1** in CDCl_3_. 9](#__RefHeading___Toc124370374)

[**Fig. S10** ^1^H NMR (600 MHz) spectrum of (*R*)-MTPA ester of **1** in CDCl_3_. 9](#__RefHeading___Toc124370375)

[**Fig. S11** HRESIMS spectrum of **2**. 10](#__RefHeading___Toc124370376)

[**Fig. S12** ^1^H NMR (600 MHz) spectrum of **2** in CDCl_3_. 10](#__RefHeading___Toc124370377)

[**Fig. S13** ^13^C NMR (150 MHz) spectrum of **2** in CDCl_3_. 11](#__RefHeading___Toc124370378)

[**Fig. S14** HSQC (600 MHz) spectrum of **2** in CDCl_3_. 11](#__RefHeading___Toc124370379)

[**Fig. S15** HMBC (600 MHz) spectrum of **2** in CDCl_3_. 12](#__RefHeading___Toc124370380)

[**Fig. S16** ^1^H-^1^H COSY (600 MHz) spectrum of **2** in CDCl_3_. 12](#__RefHeading___Toc124370381)

[**Fig. S17** UV spectrum of **2** in MeOH. 13](#__RefHeading___Toc124370382)

[**Fig. S18** IR spectrum of **2**. 13](#__RefHeading___Toc124370383)

[**Fig. S19** ^1^H NMR (600 MHz) spectrum of **2a** in CDCl_3_. 14](#__RefHeading___Toc124370384)

[**Fig. S20** ^13^C NMR (150 MHz) spectrum of **2a** in CDCl_3_. 14](#__RefHeading___Toc124370385)

[**Fig. S21** ^1^H NMR (600 MHz) spectrum of (*S*)-MTPA ester of **2a** in CDCl_3_. 15](#__RefHeading___Toc124370386)

[**Fig. S22** ^1^H NMR (600 MHz) spectrum of (*R*)-MTPA ester of **2a** in CDCl_3_. 15](#__RefHeading___Toc124370387)

[**Fig. S23** HRESIMS spectrum of **3**. 16](#__RefHeading___Toc124370388)

[**Fig. S24** ^1^H NMR (600 MHz) spectrum of **3** in MeOH-*d_4_*. 16](#__RefHeading___Toc124370389)

[**Fig. S25** ^13^C NMR (150 MHz) spectrum of **3** in MeOH-*d_4_*. 17](#__RefHeading___Toc124370390)

[**Fig. S26** HSQC (600 MHz) spectrum of **3** in MeOH-*d_4_*. 17](#__RefHeading___Toc124370391)

[**Fig. S27** HMBC (600 MHz) spectrum of **3** in MeOH-*d_4_*. 18](#__RefHeading___Toc124370392)

[**Fig. S28** ^1^H-^1^H COSY (600 MHz) spectrum of **3** in MeOH-*d_4_*. 18](#__RefHeading___Toc124370393)

[**Fig. S29** UV spectrum of **3** in MeOH. 19](#__RefHeading___Toc124370394)

[**Fig. S30** IR spectrum of **3**. 19](#__RefHeading___Toc124370395)

[**Fig. S31** HRESIMS spectrum of **4**. 20](#__RefHeading___Toc124370396)

[**Fig. S32** ^1^H NMR (600 MHz) spectrum of **4** in CDCl_3_. 20](#__RefHeading___Toc124370397)

[**Fig. S33** ^13^C NMR (150 MHz) spectrum of **4** in CDCl_3_. 21](#__RefHeading___Toc124370398)

[**Fig. S34** HSQC (600 MHz) spectrum of **4** in CDCl_3_. 21](#__RefHeading___Toc124370399)

[**Fig. S35** HMBC (600 MHz) spectrum of **4** in CDCl_3_. 22](#__RefHeading___Toc124370400)

[**Fig. S36** ^1^H-^1^H COSY (600 MHz) spectrum of **4** in CDCl_3_. 22](#__RefHeading___Toc124370401)

[**Fig. S37** UV spectrum of **4** in MeOH. 23](#__RefHeading___Toc124370402)

[**Fig. S38** IR spectrum of **4**. 23](#__RefHeading___Toc124370403)

[**Fig. S39** HRESIMS spectrum of **5**. 24](#__RefHeading___Toc124370404)

[**Fig. S40** ^1^H NMR (600 MHz) spectrum of **5** in CDCl_3_. 24](#__RefHeading___Toc124370405)

[**Fig. S41** ^13^C NMR (150 MHz) spectrum of **5** in CDCl_3_. 25](#__RefHeading___Toc124370406)

[**Fig. S42** HSQC (600 MHz) spectrum of **5** in CDCl_3_. 25](#__RefHeading___Toc124370407)

[**Fig. S43** HMBC (600 MHz) spectrum of **5** in CDCl_3_. 26](#__RefHeading___Toc124370408)

[**Fig. S44** ^1^H-^1^H COSY (600 MHz) spectrum of **5** in CDCl_3_. 26](#__RefHeading___Toc124370409)

[**Fig. S45** UV spectrum of **5** in MeOH. 27](#__RefHeading___Toc124370410)

[**Fig. S46** IR spectrum of **5**. 27](#__RefHeading___Toc124370411)

[**Fig. S47** HRESIMS spectrum of **6**. 28](#__RefHeading___Toc124370412)

[**Fig. S48** ^1^H NMR (600 MHz) spectrum of **6** in MeOH-*d_4_*. 28](#__RefHeading___Toc124370413)

[**Fig. S49** ^13^C NMR (150 MHz) spectrum of **6** in MeOH-*d_4_*. 29](#__RefHeading___Toc124370414)

[**Fig. S50** HSQC (600 MHz) spectrum of **6** in MeOH-*d_4_*. 29](#__RefHeading___Toc124370415)

[**Fig. S51** HMBC (600 MHz) spectrum of **6** in MeOH-*d_4_*. 30](#__RefHeading___Toc124370416)

[**Fig. S52** UV spectrum of **6** in MeOH. 30](#__RefHeading___Toc124370417)

[**Fig. S53** IR spectrum of **6**. 31](#__RefHeading___Toc124370418)

[**Fig. S54** HRESIMS spectrum of **7**. 31](#__RefHeading___Toc124370419)

[**Fig. S55** ^1^H NMR (600 MHz) spectrum of **7** in MeOH-*d_4_*. 32](#__RefHeading___Toc124370420)

[**Fig. S56** ^13^C NMR (150 MHz) spectrum of **7** in MeOH-*d_4_*. 32](#__RefHeading___Toc124370421)

[**Fig. S57** HSQC (600 MHz) spectrum of **7** in MeOH-*d_4_*. 33](#__RefHeading___Toc124370422)

[**Fig. S58** HMBC (600 MHz) spectrum of **7** in MeOH-*d_4_*. 33](#__RefHeading___Toc124370423)

[**Fig. S59** UV spectrum of **7** in MeOH. 34](#__RefHeading___Toc124370424)

[**Fig. S60** IR spectrum of **7**. 34](#__RefHeading___Toc124370425)

[**Fig. S61** HRESIMS spectrum of **8**. 35](#__RefHeading___Toc124370426)

[**Fig. S62** ^1^H NMR (600 MHz) spectrum of **8** in MeOH-*d_4_*. 35](#__RefHeading___Toc124370427)

[**Fig. S63** ^13^C NMR (150 MHz) spectrum of **8** in MeOH-*d_4_*. 36](#__RefHeading___Toc124370428)

[**Fig. S64** HSQC (600 MHz) spectrum of **8** in MeOH-*d_4_*. 36](#__RefHeading___Toc124370429)

[**Fig. S65** HMBC (600 MHz) spectrum of **8** in MeOH-*d_4_*. 37](#__RefHeading___Toc124370430)

[**Fig. S66** UV spectrum of **8** in MeOH. 37](#__RefHeading___Toc124370431)

[**Fig. S67** IR spectrum of **8**. 38](#__RefHeading___Toc124370432)

[**Fig. S68** The proposed biogenetic relationship of the isolated metabolites 38](#__RefHeading___Toc124370433)

[The experimental procedure of alkaline hydrolysis and Mosher’ method 39](#__RefHeading___Toc124370434)

[Antimicrobial Assays 39](#__RefHeading___Toc124370435)

[Table S1 The inhibitory activity against Gram-negative bacteria of compounds 1-24 40](#__RefHeading___Toc124370436)

[Table S2 The inhibitory activity against Gram-positive bacteria, Mycobacterium and Fungus of compounds 1-24 41](#__RefHeading___Toc124370437)

[Cytotoxicity Assay 42](#__RefHeading___Toc124370438)

[Table S3 The initial screening inhibition rate of the tested compounds 43](#__RefHeading___Toc124370439)


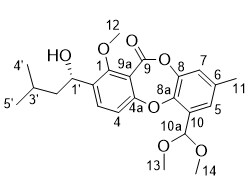


**Fig. S1** HRESIMS spectrum of **1**.


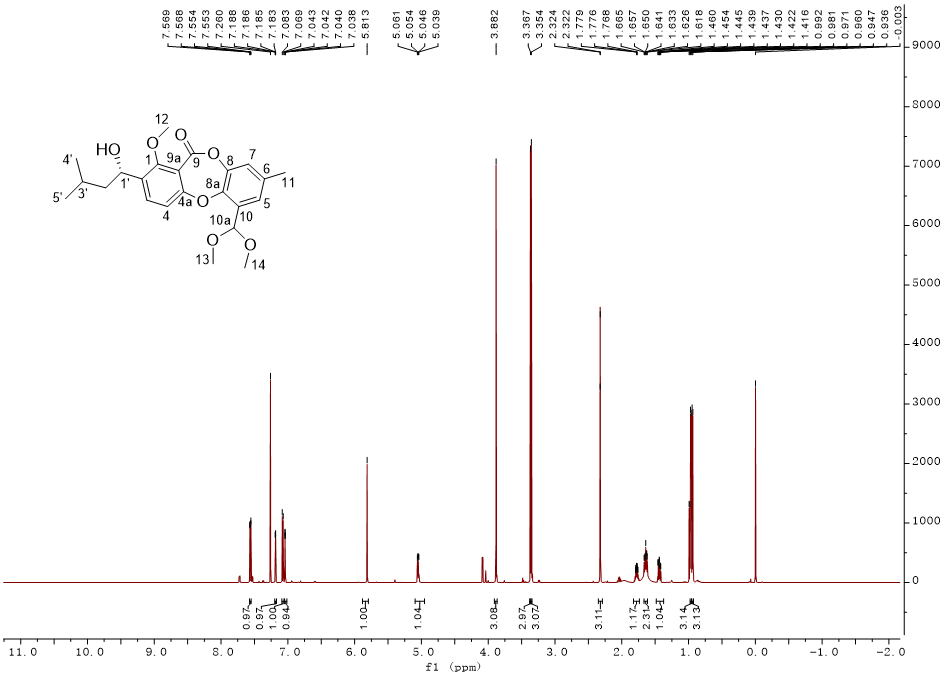


**Fig. S2** ^1^H NMR (600 MHz) spectrum of **1** in CDCl_3_**.**


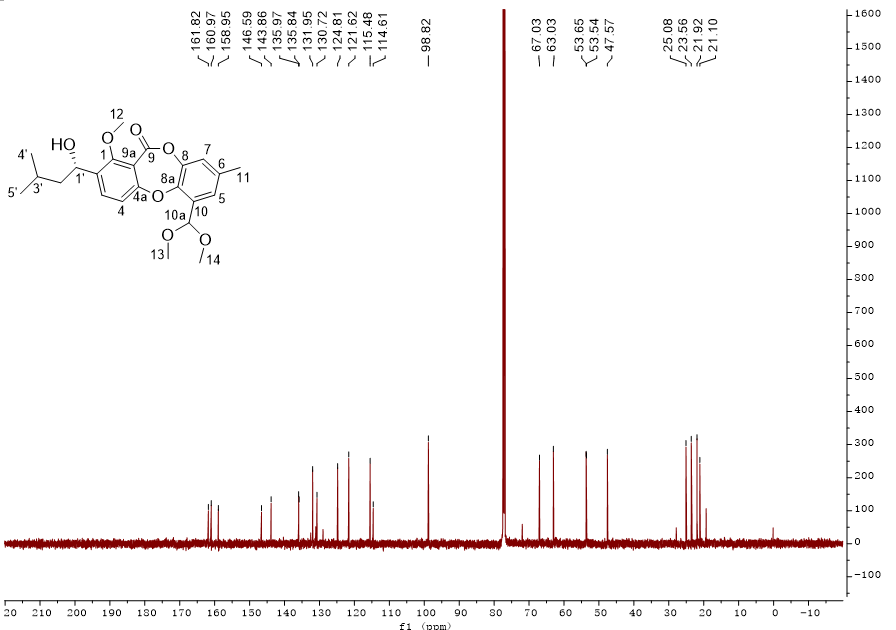


**Fig. S3** ^13^C NMR (150 MHz) spectrum of **1** in CDCl_3_**.**


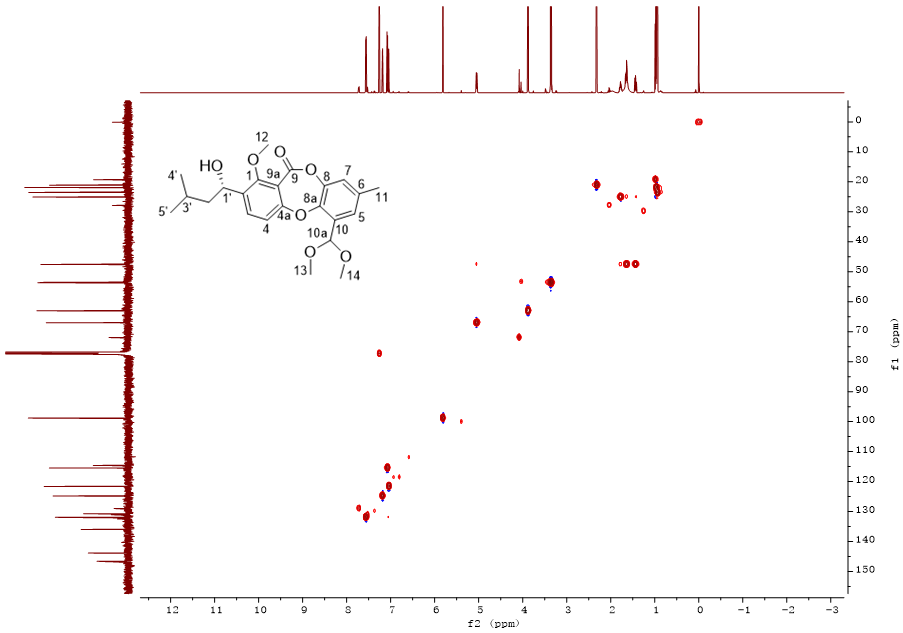


**Fig. S4** HSQC (600 MHz) spectrum of **1** in CDCl_3_.


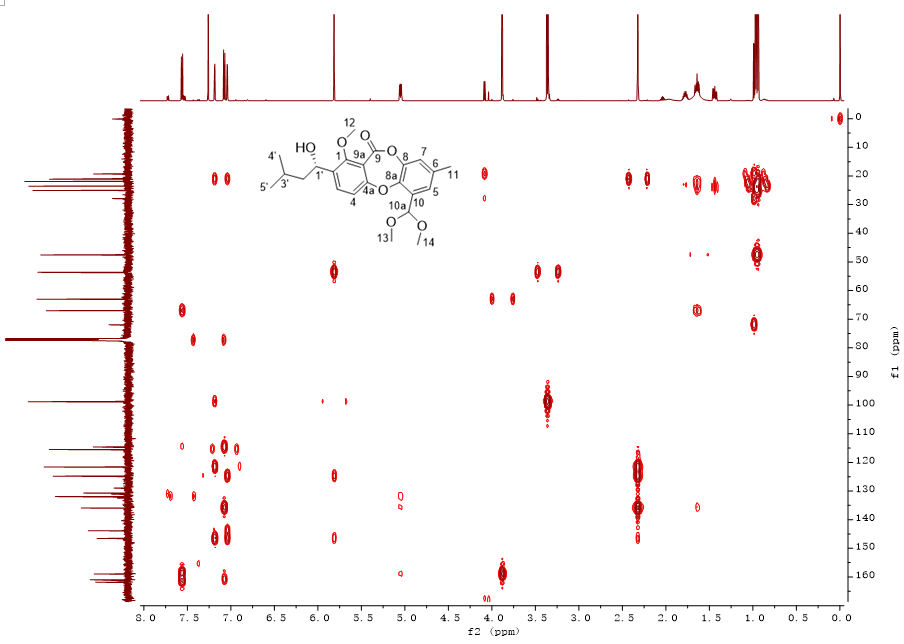


**Fig. S5** HMBC (600 MHz) spectrum of **1** in CDCl_3_.


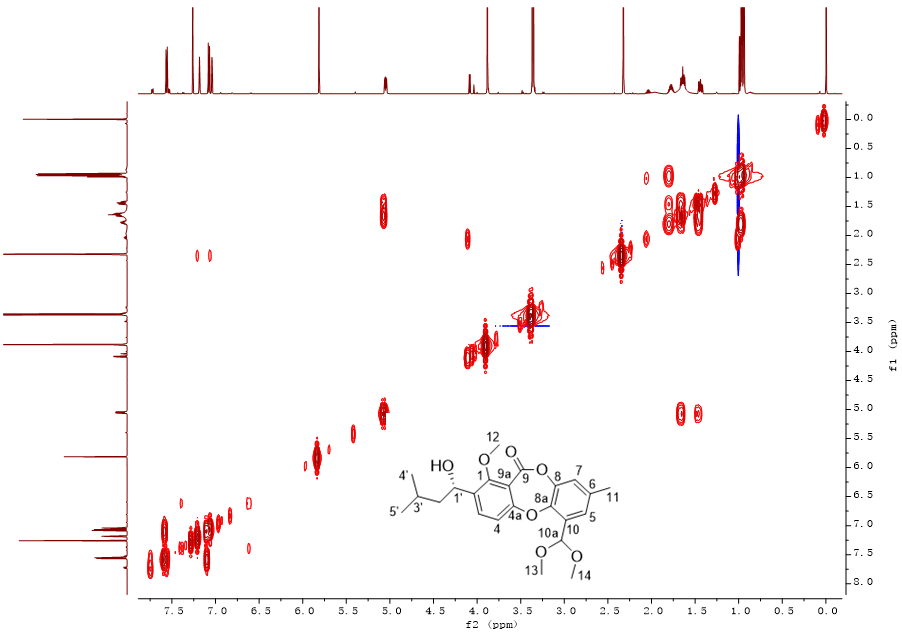


**Fig. S6** ^1^H-^1^H COSY (600 MHz) spectrum of **1** in CDCl_3_.


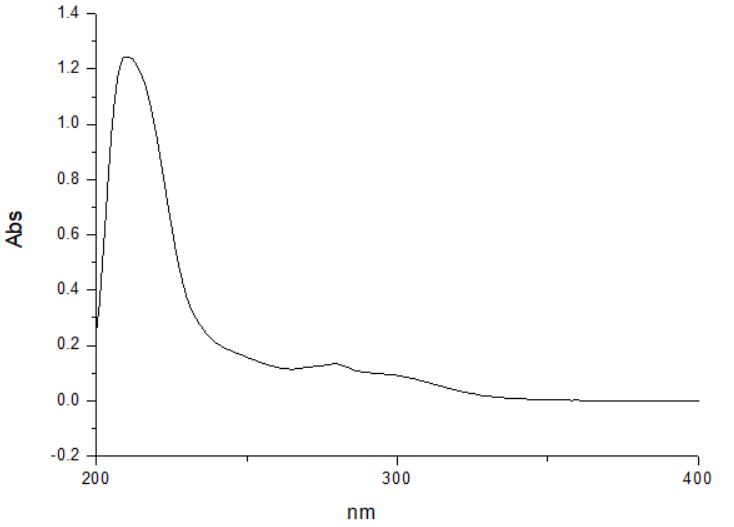


**Fig. S7** UV spectrum of **1** in MeOH.


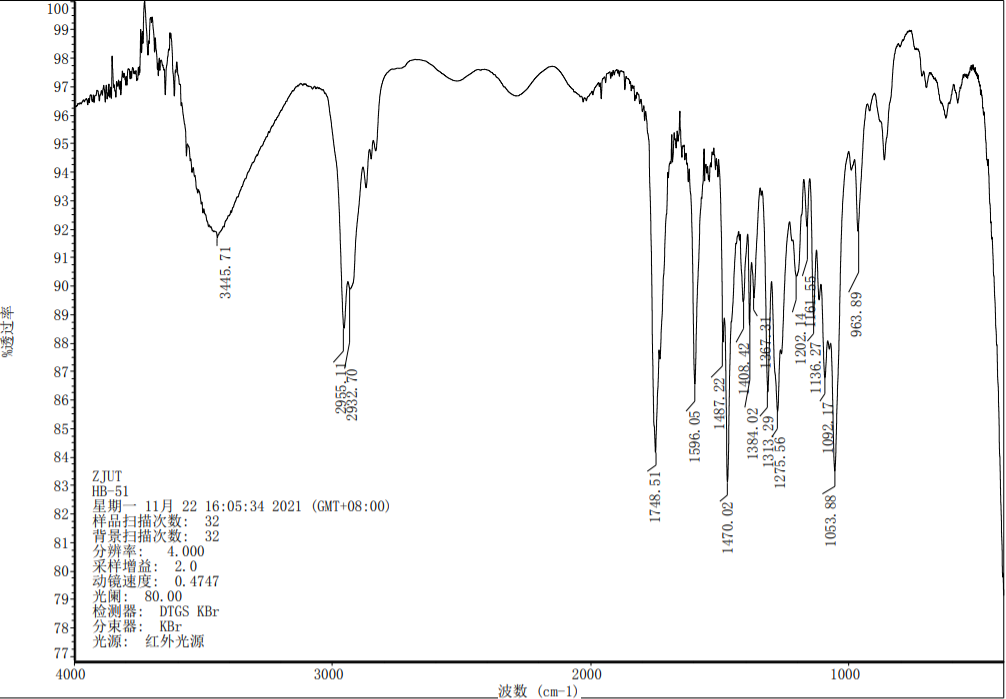


**Fig. S8** IR spectrum of **1**.


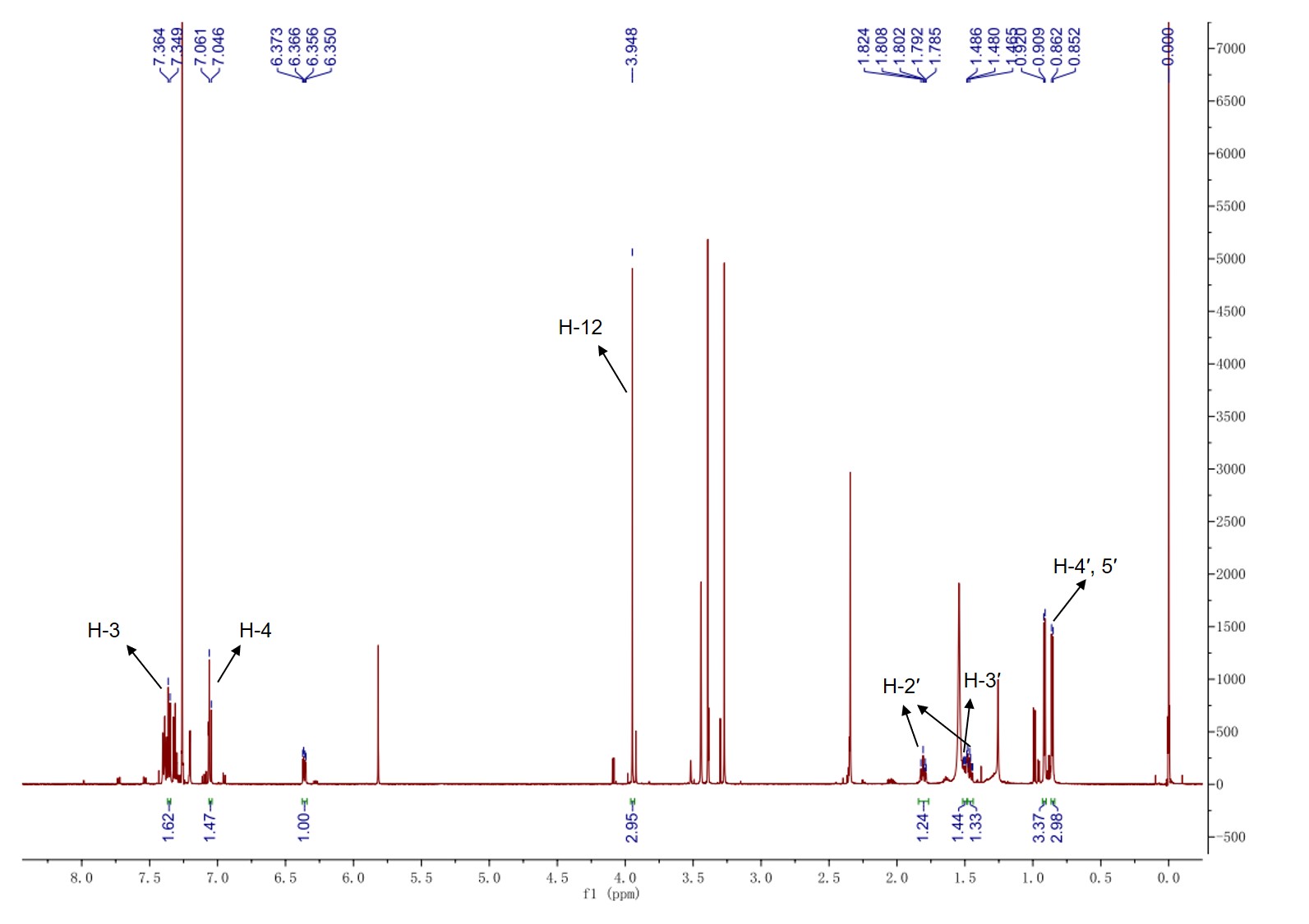


**Fig. S9** ^1^H NMR (600 MHz) spectrum of (*S*)-MTPA ester of **1** in CDCl_3_.


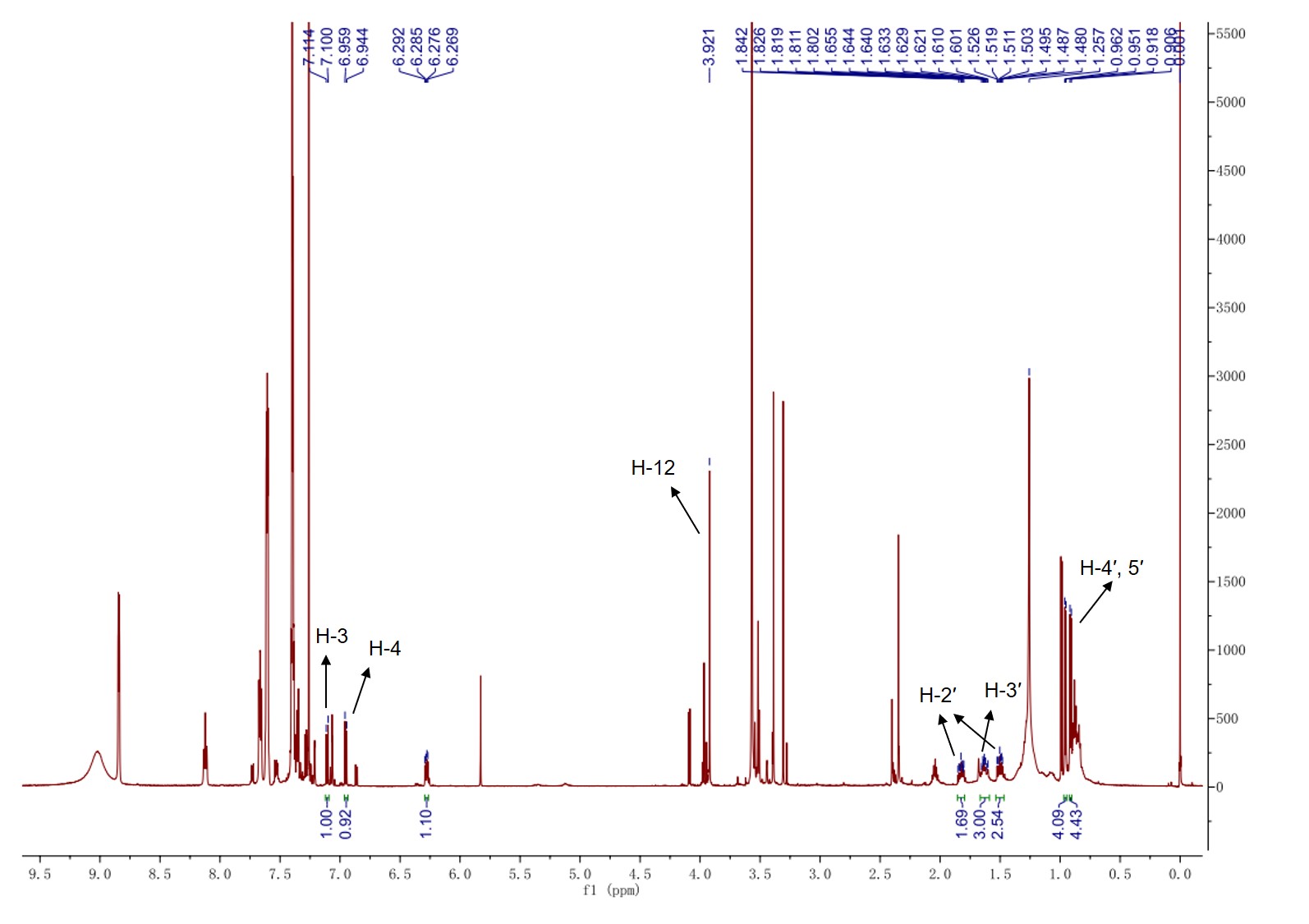


**Fig. S10** ^1^H NMR (600 MHz) spectrum of (*R*)-MTPA ester of **1** in CDCl_3_.


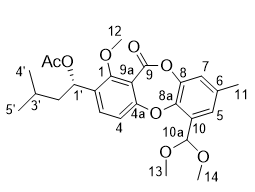


**Fig. S11** HRESIMS spectrum of **2**.


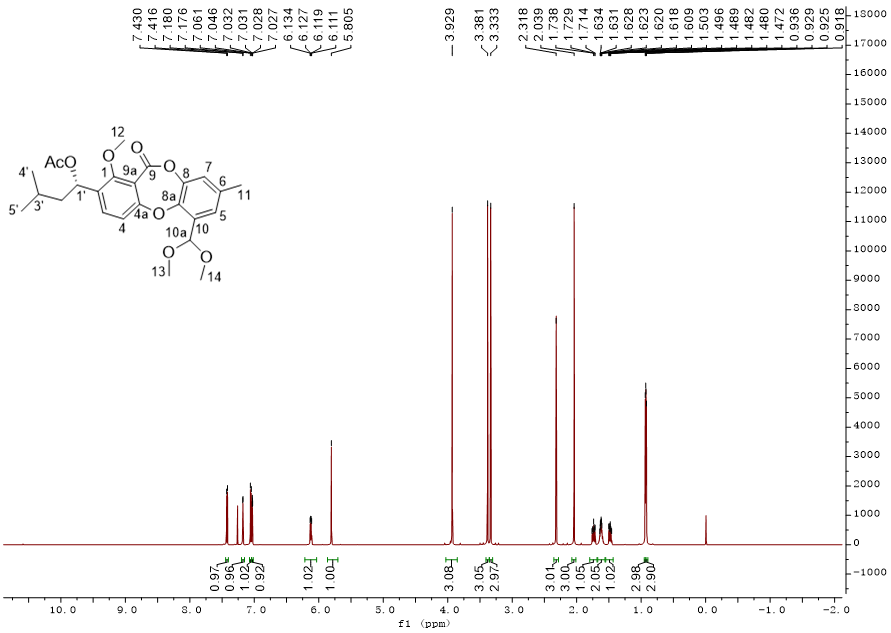


**Fig. S12** ^1^H NMR (600 MHz) spectrum of **2** in CDCl_3_.


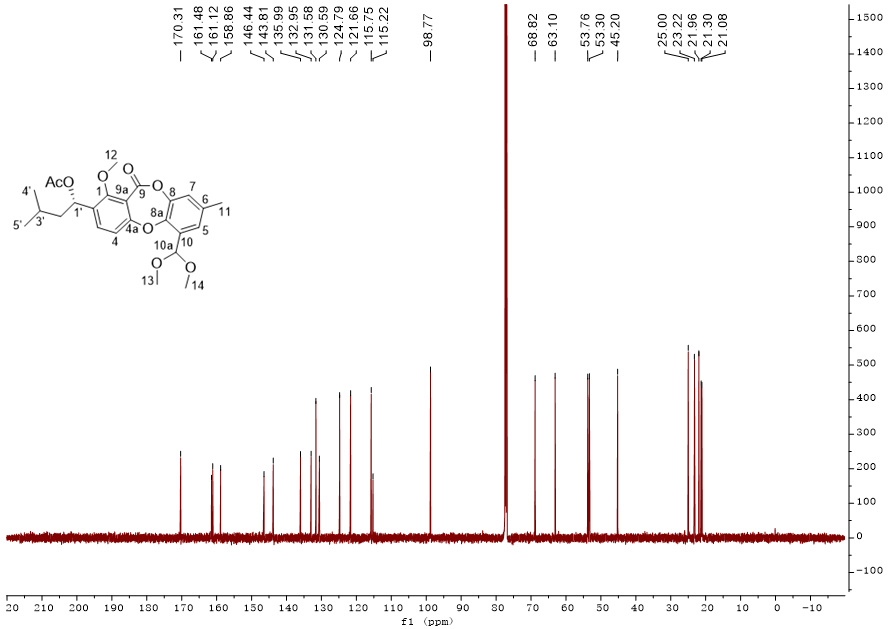


**Fig. S13** ^13^C NMR (150 MHz) spectrum of **2** in CDCl_3_.


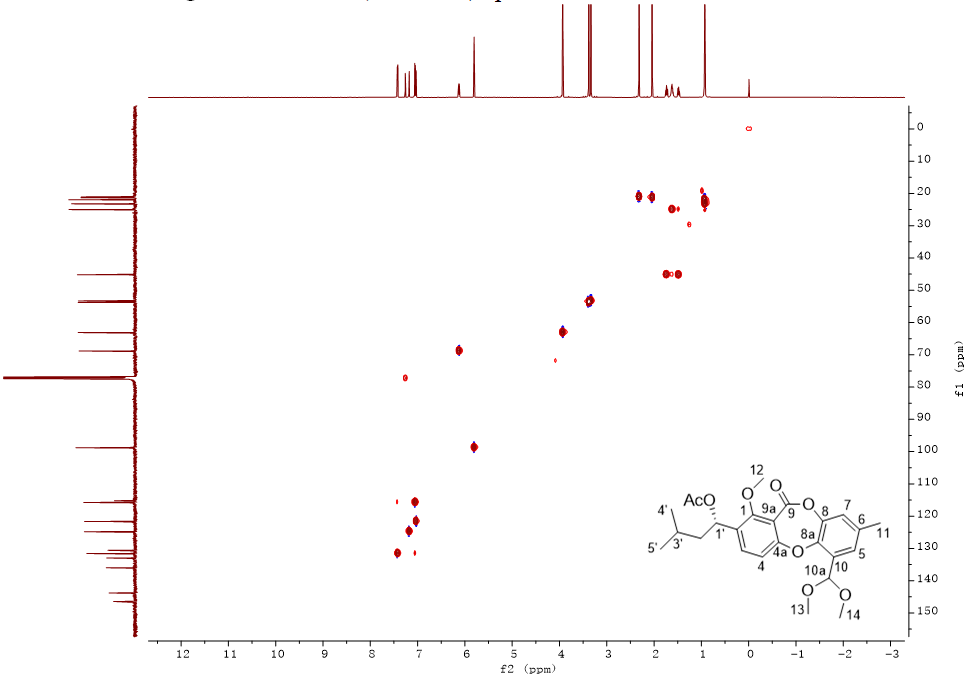


**Fig. S14** HSQC (600 MHz) spectrum of **2** in CDCl_3_.


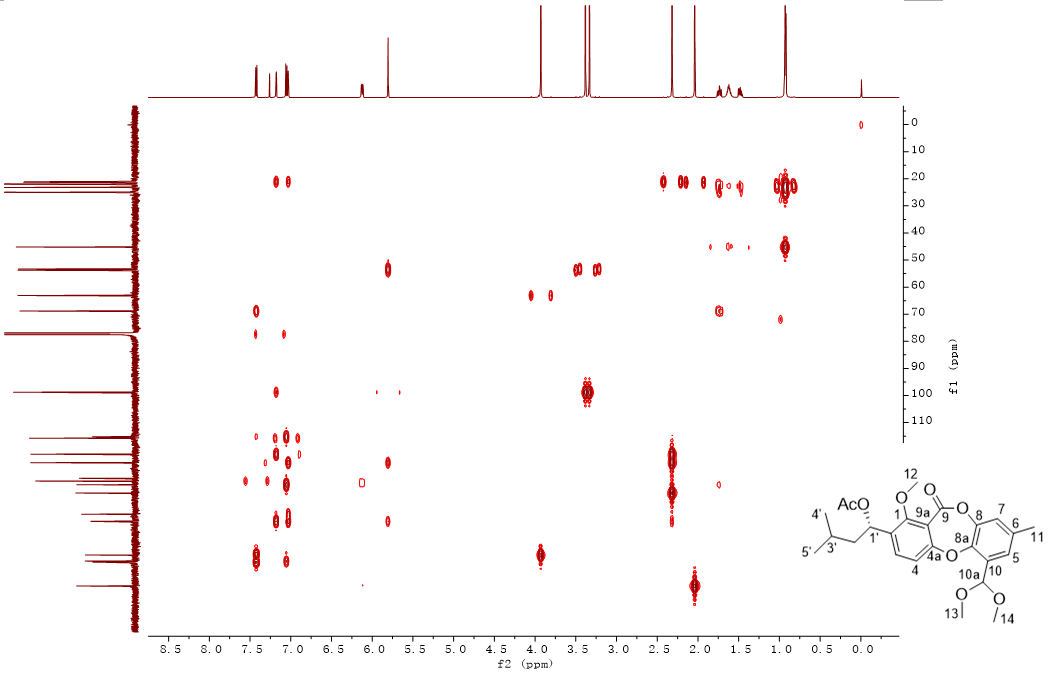


**Fig. S15** HMBC (600 MHz) spectrum of **2** in CDCl_3_.


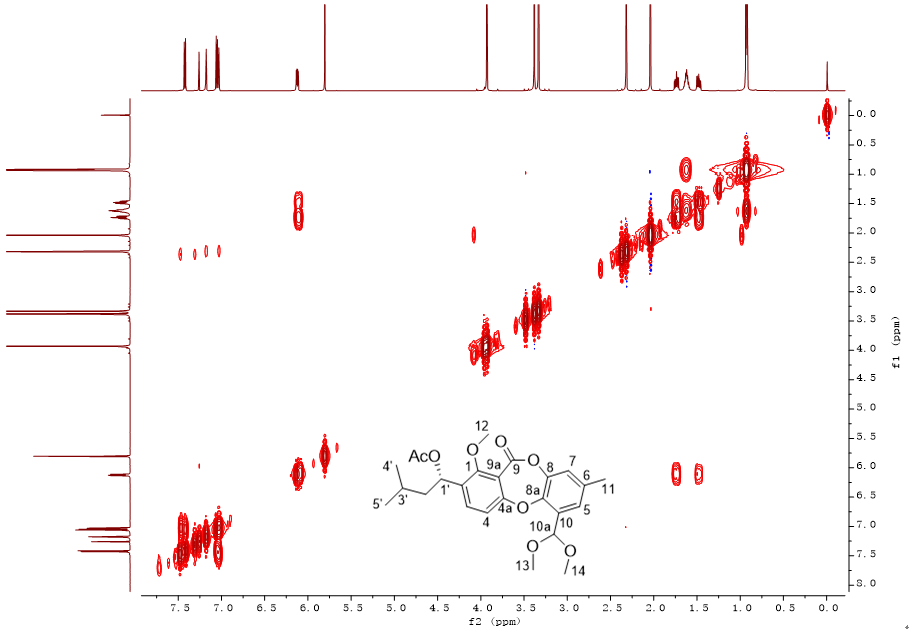


**Fig. S16** ^1^H-^1^H COSY (600 MHz) spectrum of **2** in CDCl_3_.


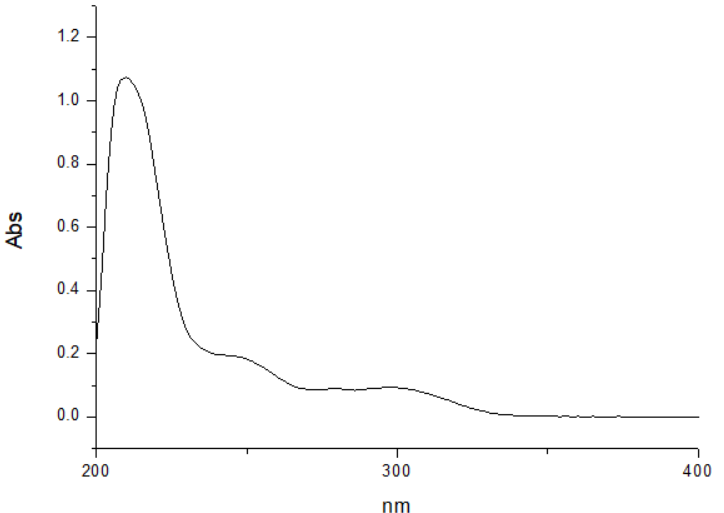


**Fig. S17** UV spectrum of **2** in MeOH.


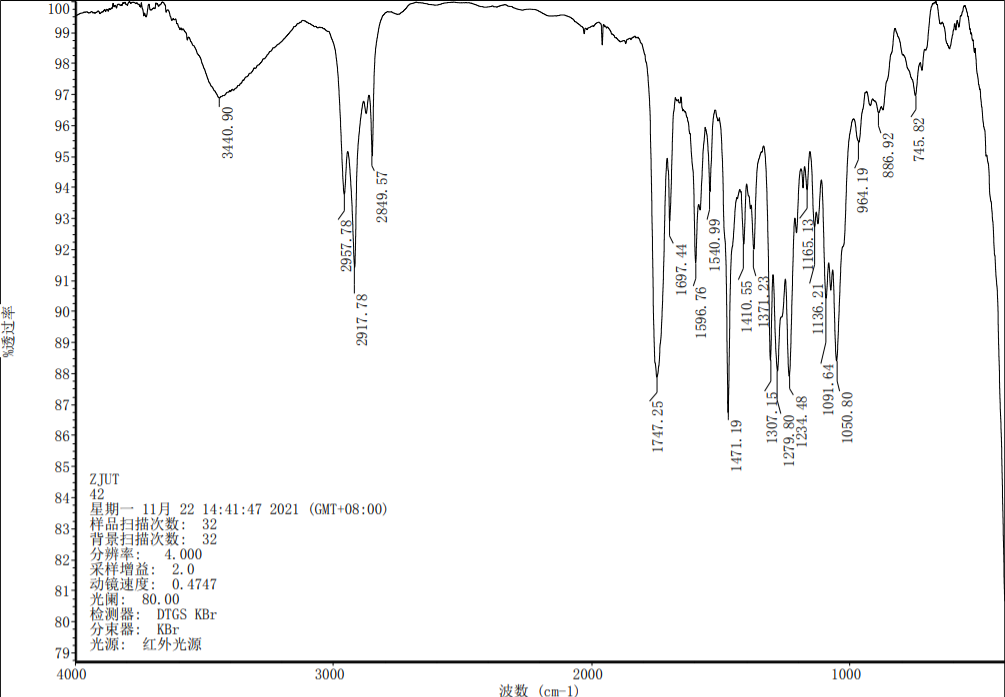


**Fig. S18** IR spectrum of **2**.


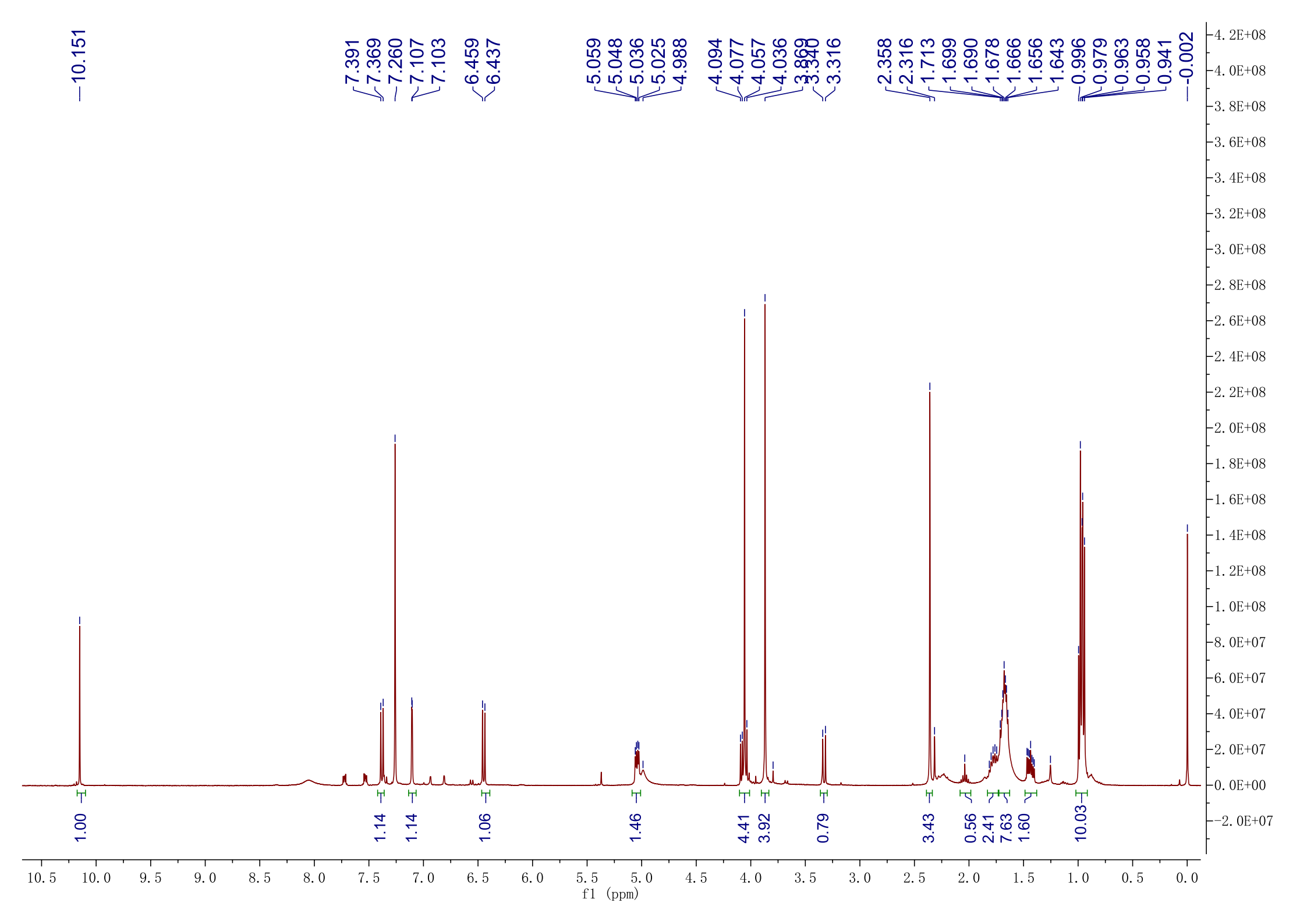


**Fig. S19** ^1^H NMR (600 MHz) spectrum of **2a** in CDCl_3_.


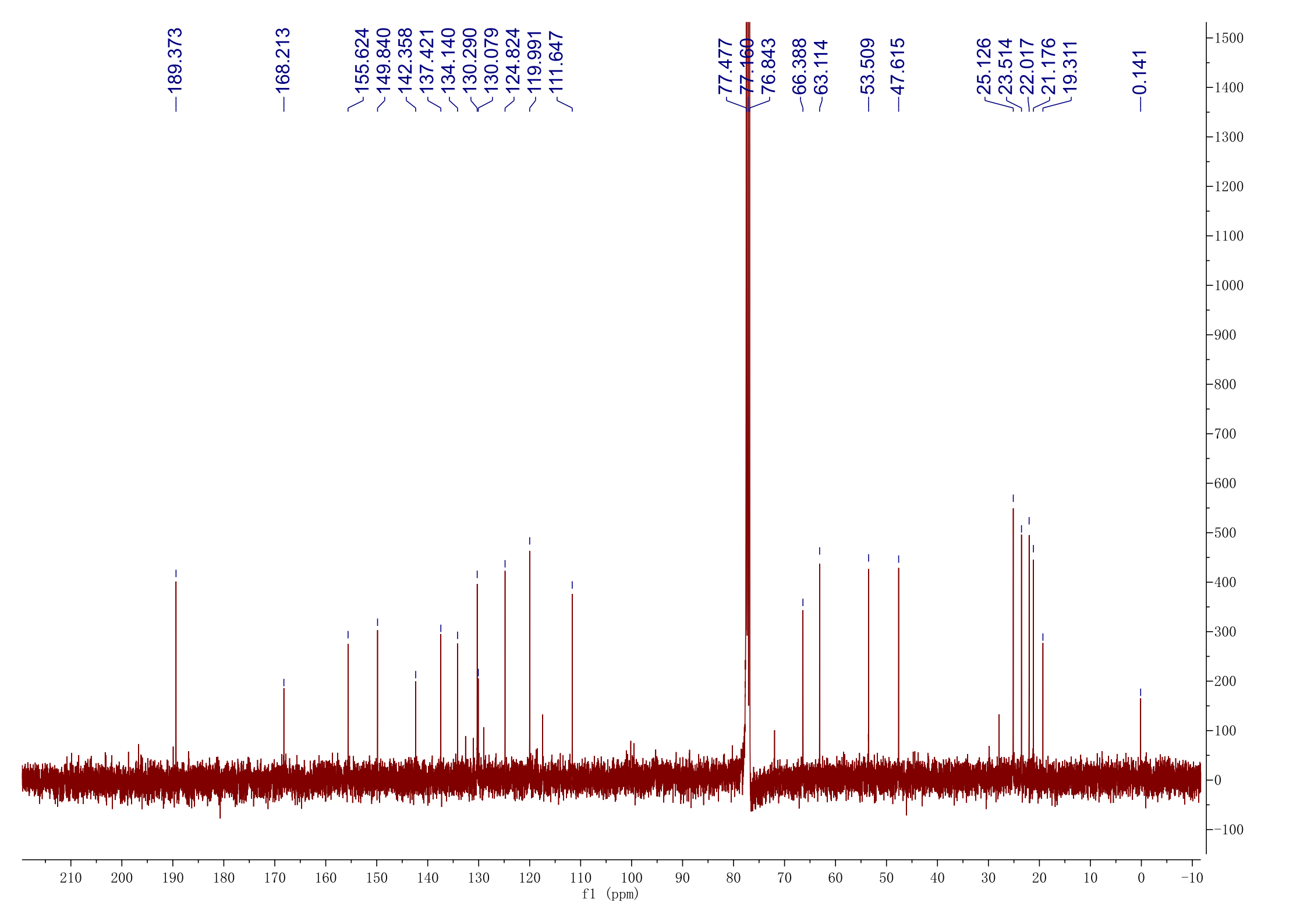


**Fig. S20** ^13^C NMR (150 MHz) spectrum of **2a** in CDCl_3_.


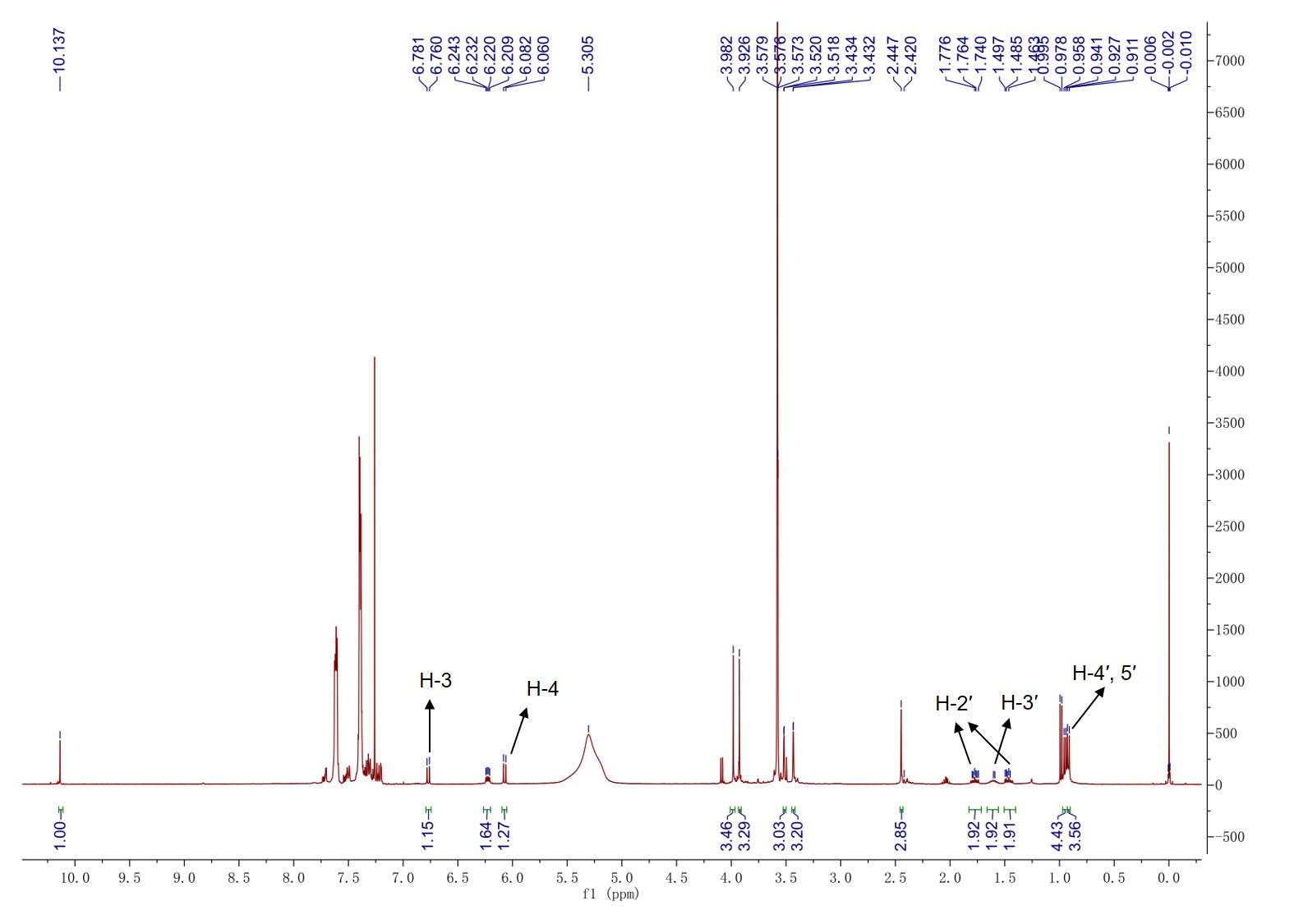


**Fig. S21** ^1^H NMR (600 MHz) spectrum of (*S*)-MTPA ester of **2a** in CDCl_3_.


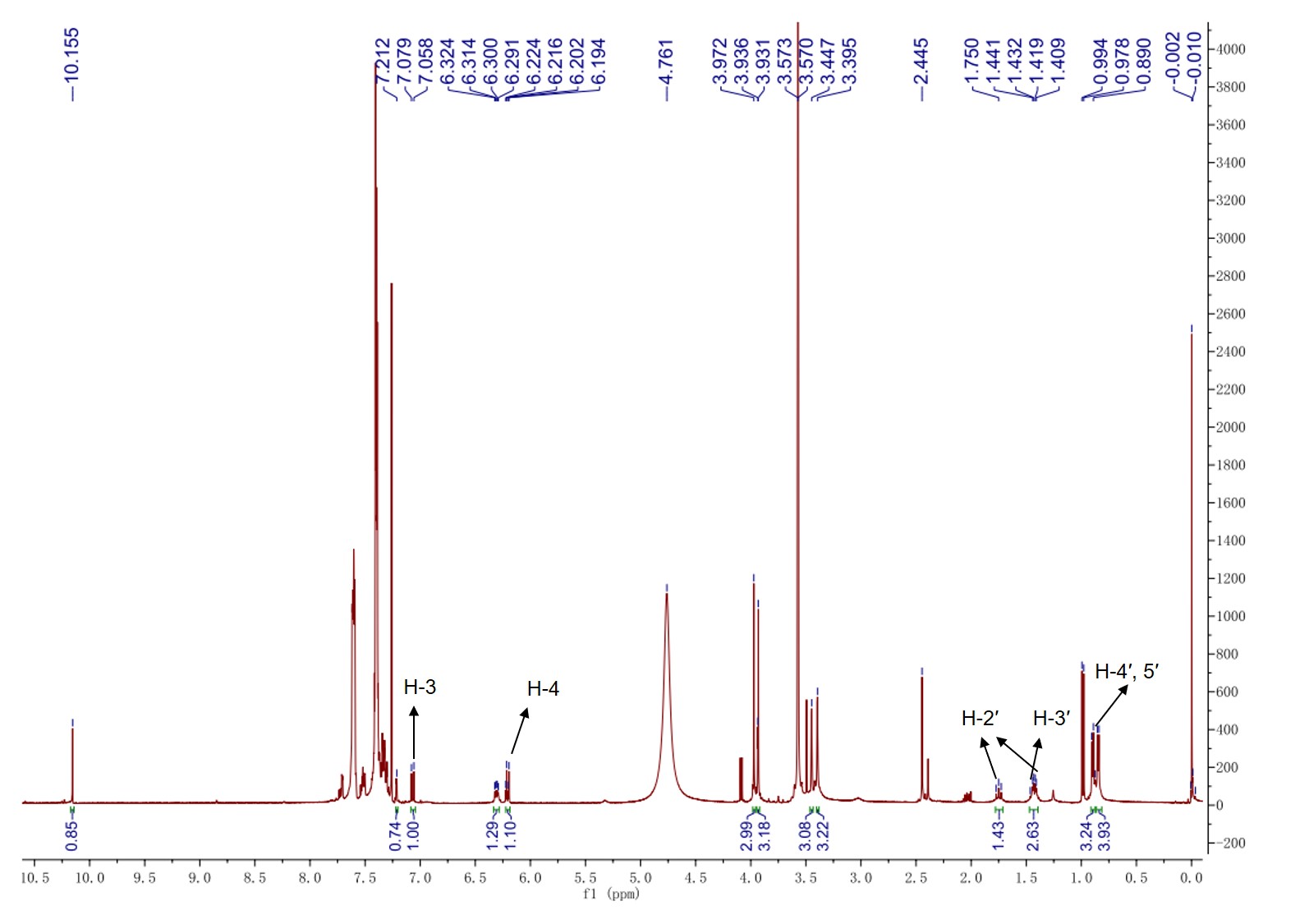


**Fig. S22** ^1^H NMR (600 MHz) spectrum of (*R*)-MTPA ester of **2a** in CDCl_3_.


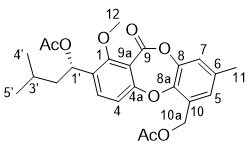


**Fig. S23** HRESIMS spectrum of **3**.


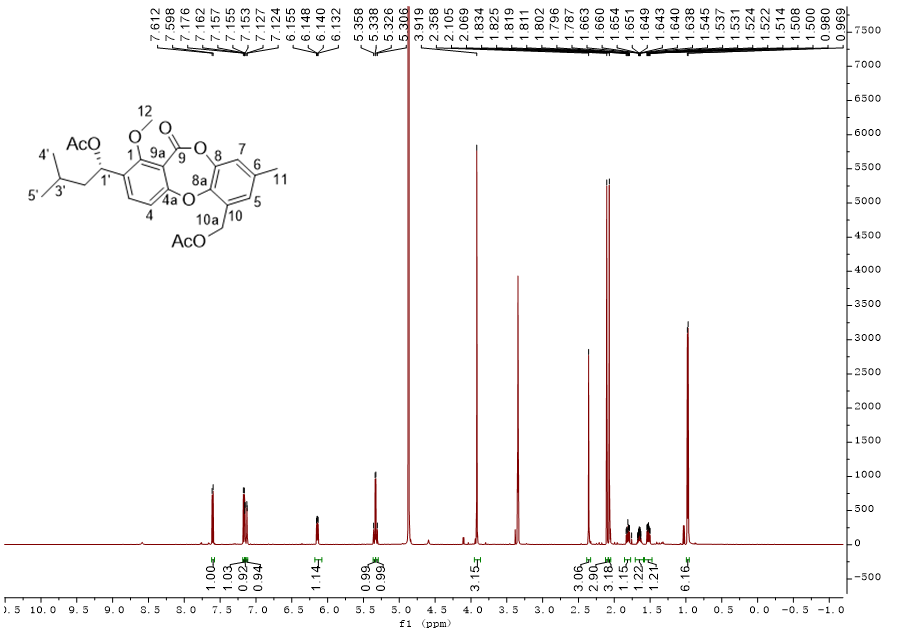


**Fig. S24** ^1^H NMR (600 MHz) spectrum of **3** in MeOH-*d_4_*.


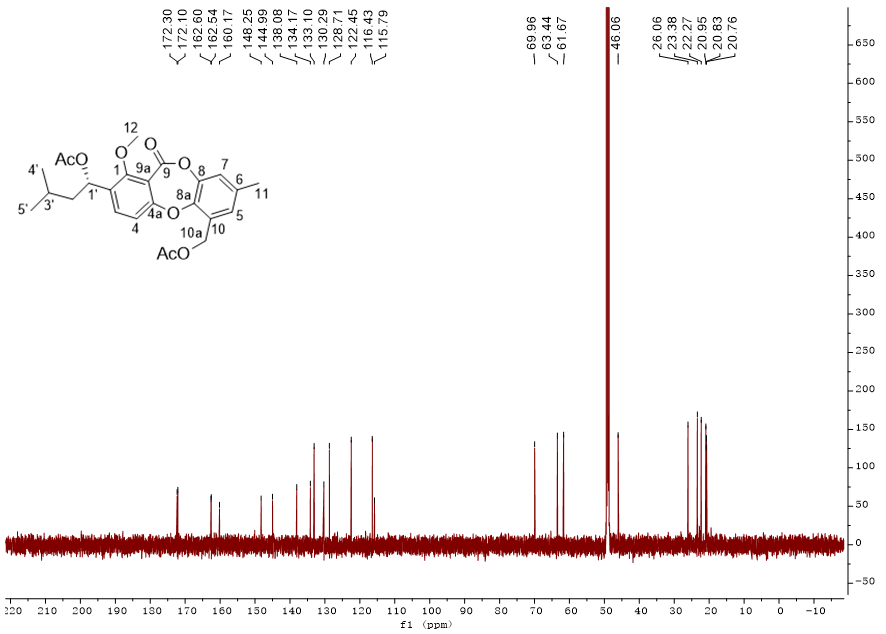


**Fig. S25** ^13^C NMR (150 MHz) spectrum of **3** in MeOH-*d_4_*.


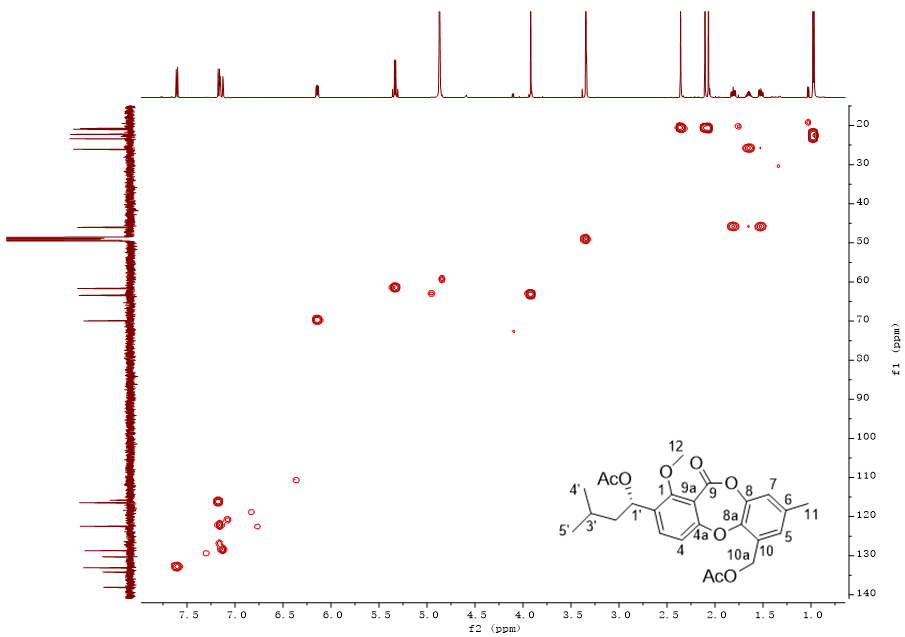


**Fig. S26** HSQC (600 MHz) spectrum of **3** in MeOH-*d_4_*.


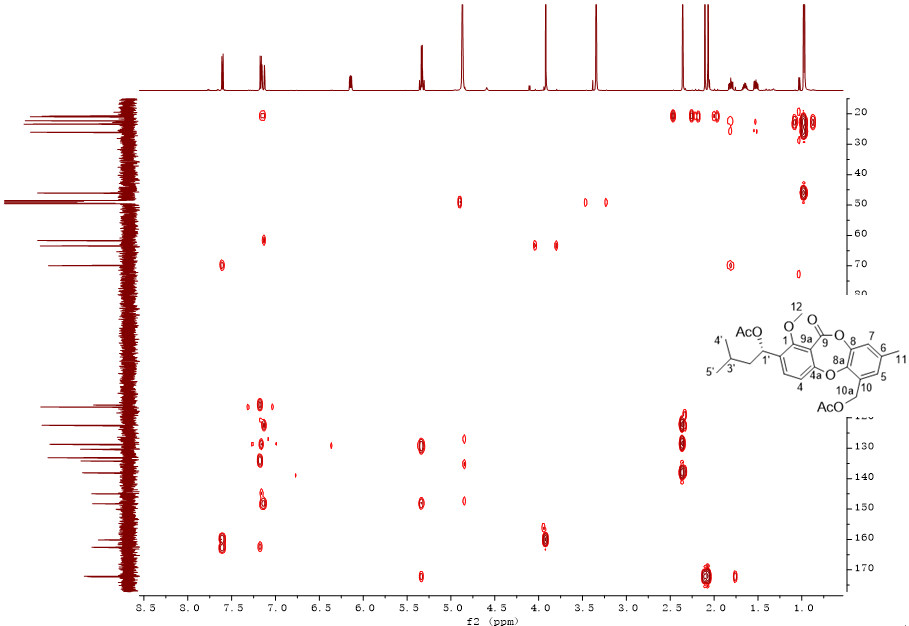


**Fig. S27** HMBC (600 MHz) spectrum of **3** in MeOH-*d_4_*.


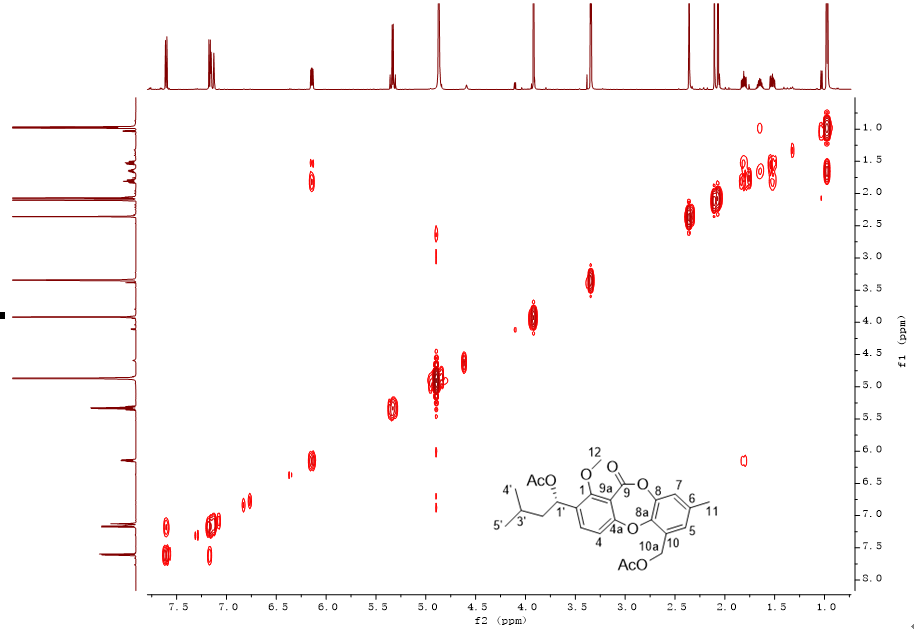


**Fig. S28** ^1^H-^1^H COSY (600 MHz) spectrum of **3** in MeOH-*d_4_*.


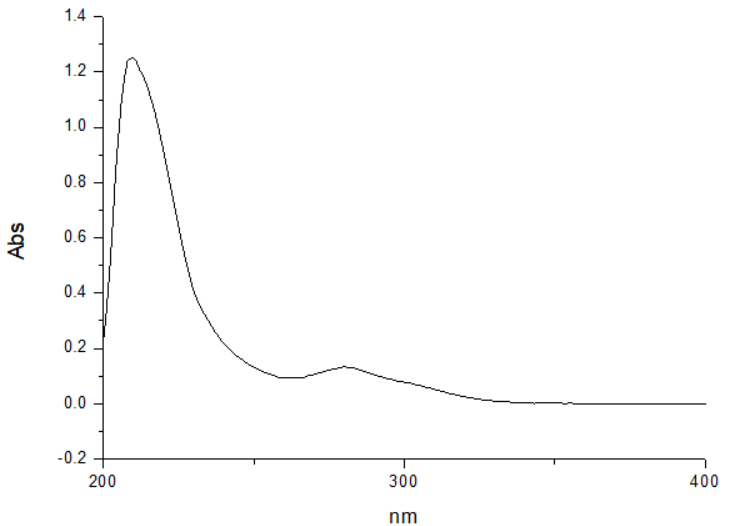


**Fig. S29** UV spectrum of **3** in MeOH.


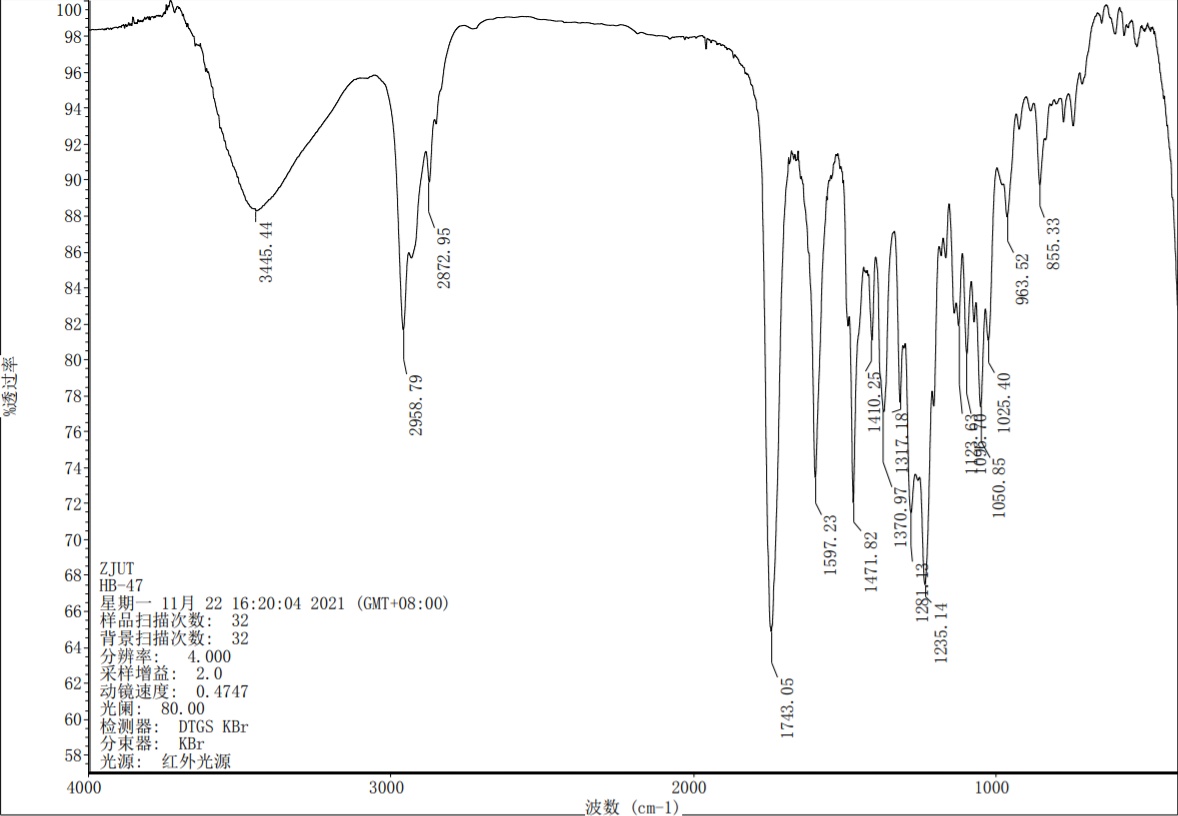


**Fig. S30** IR spectrum of **3**.


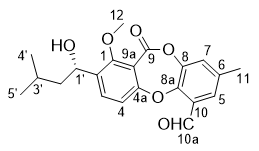


**Fig. S31** HRESIMS spectrum of **4**.


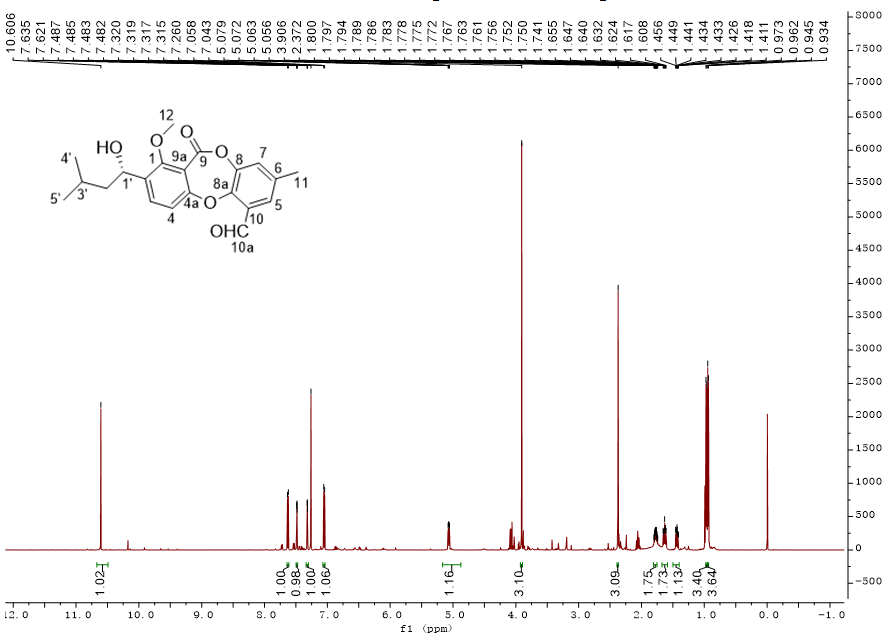


**Fig. S32** ^1^H NMR (600 MHz) spectrum of **4** in CDCl_3_.


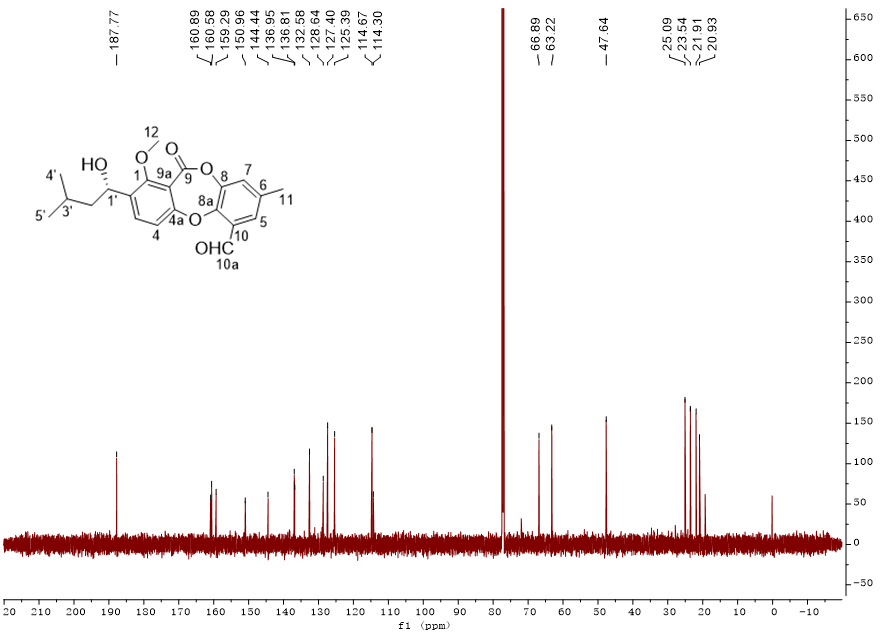


**Fig. S33** ^13^C NMR (150 MHz) spectrum of **4** in CDCl_3_.


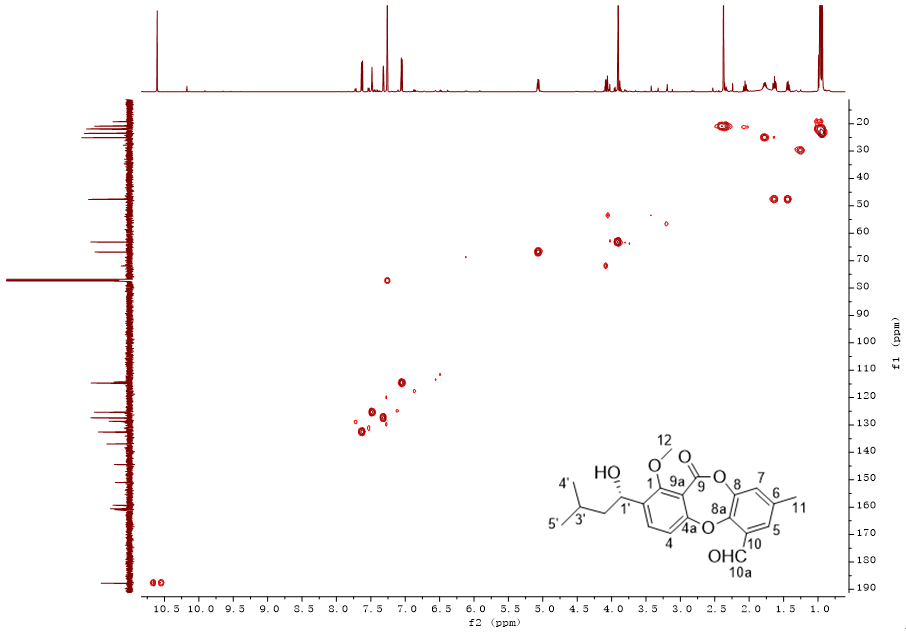


**Fig. S34** HSQC (600 MHz) spectrum of **4** in CDCl_3_.


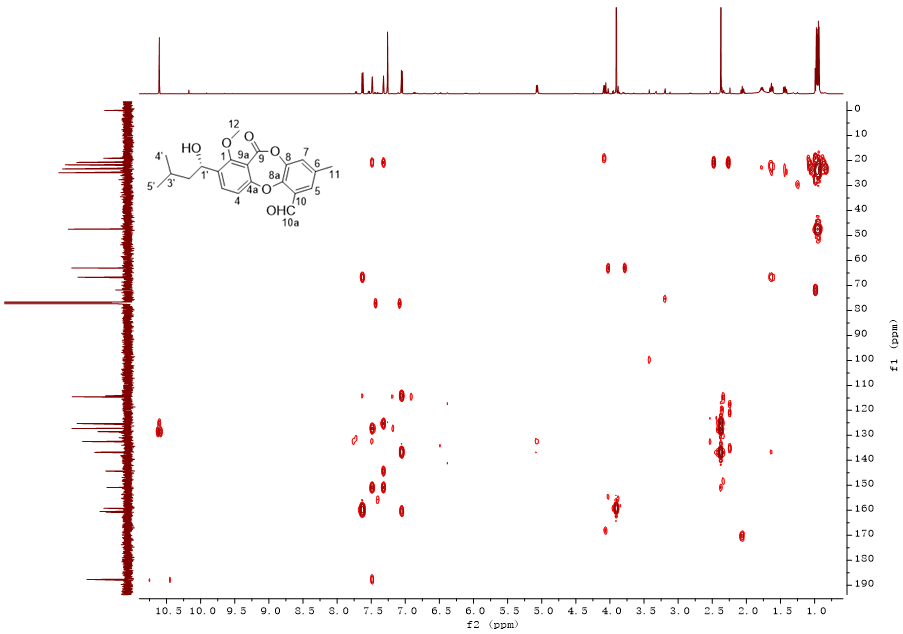


**Fig. S35** HMBC (600 MHz) spectrum of **4** in CDCl_3_.


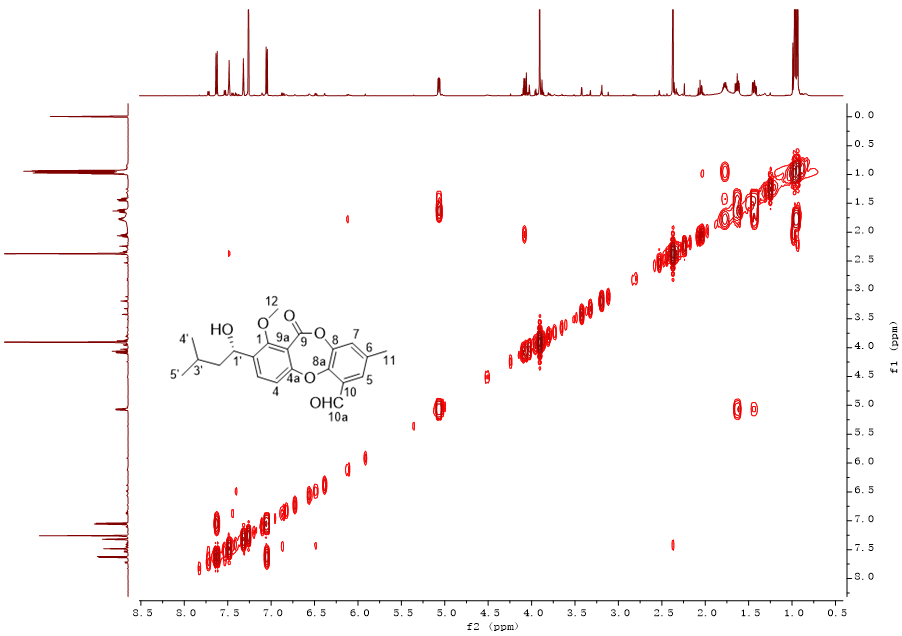


**Fig. S36** ^1^H-^1^H COSY (600 MHz) spectrum of **4** in CDCl_3_.


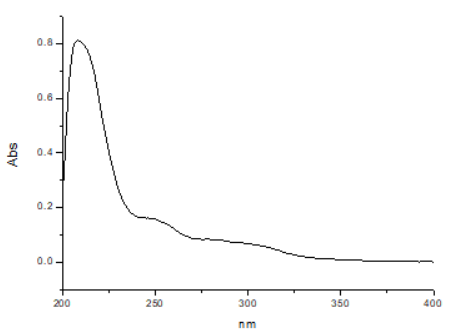


**Fig. S37** UV spectrum of **4** in MeOH.


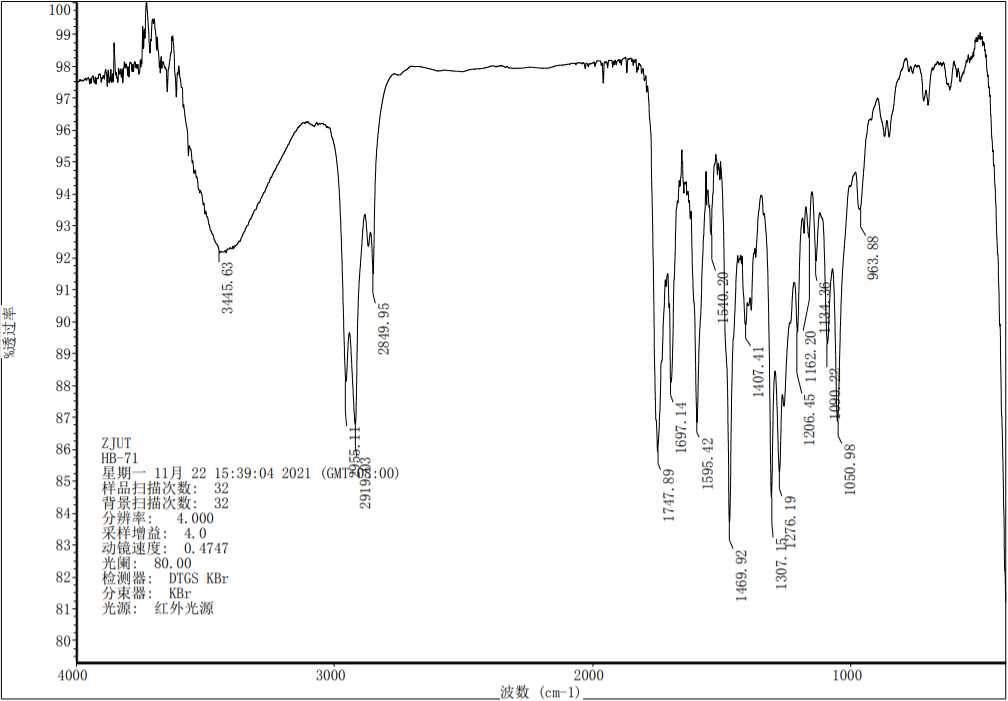


**Fig. S38** IR spectrum of **4**.


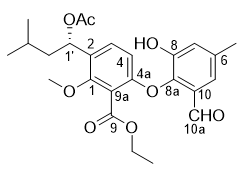


**Fig. S39** HRESIMS spectrum of **5**.


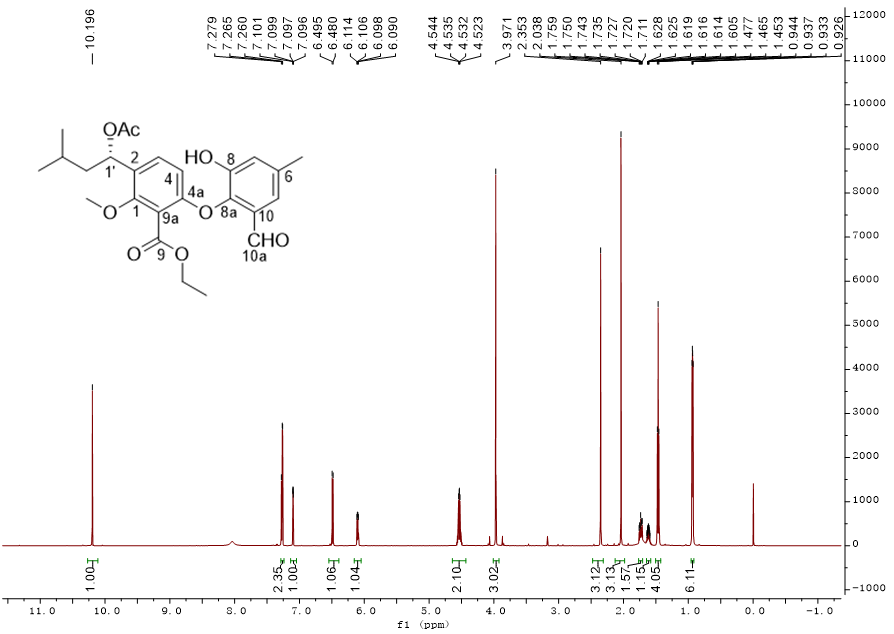


**Fig. S40** ^1^H NMR (600 MHz) spectrum of **5** in CDCl_3_.


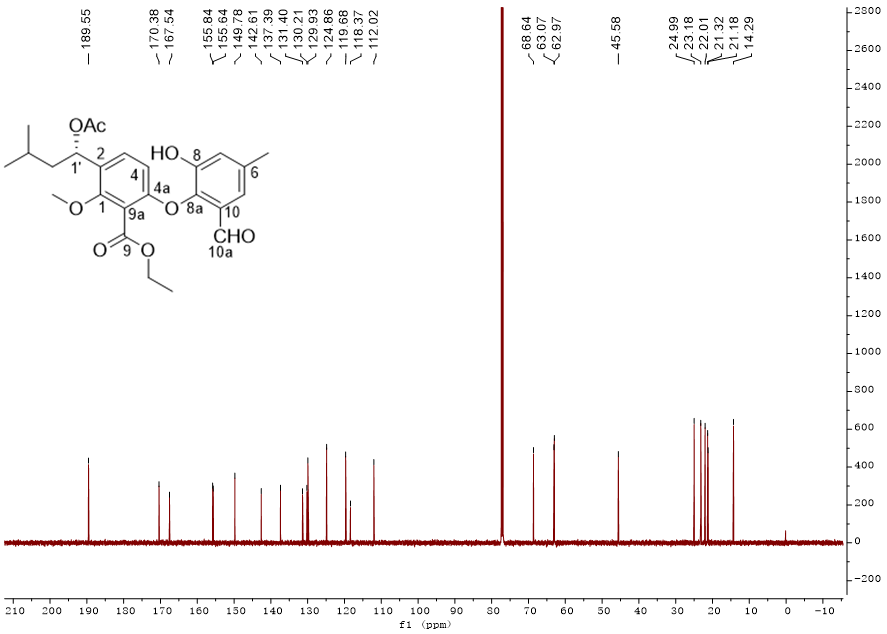


**Fig. S41** ^13^C NMR (150 MHz) spectrum of **5** in CDCl_3_.


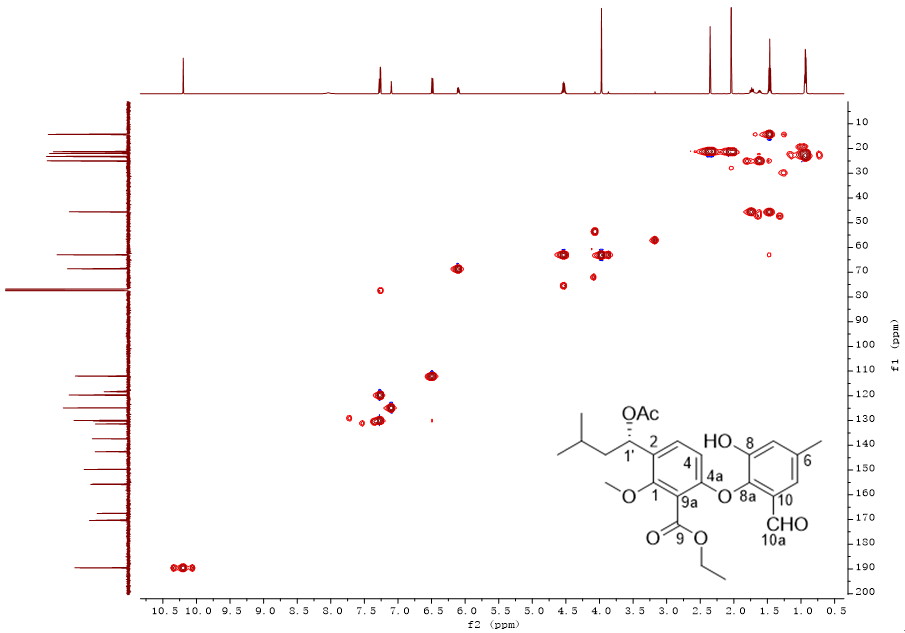


**Fig. S42** HSQC (600 MHz) spectrum of **5** in CDCl_3_.


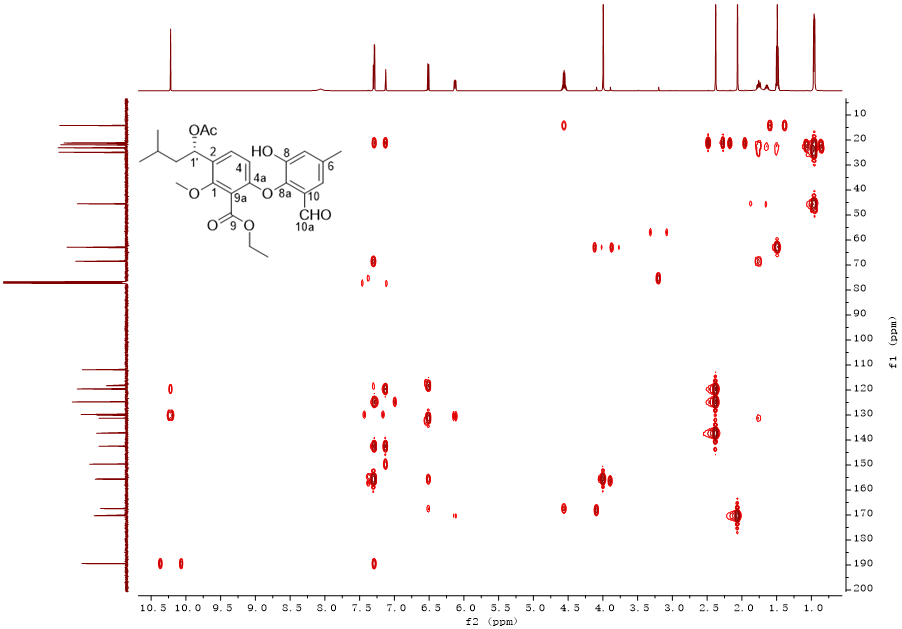


**Fig. S43** HMBC (600 MHz) spectrum of **5** in CDCl_3_.


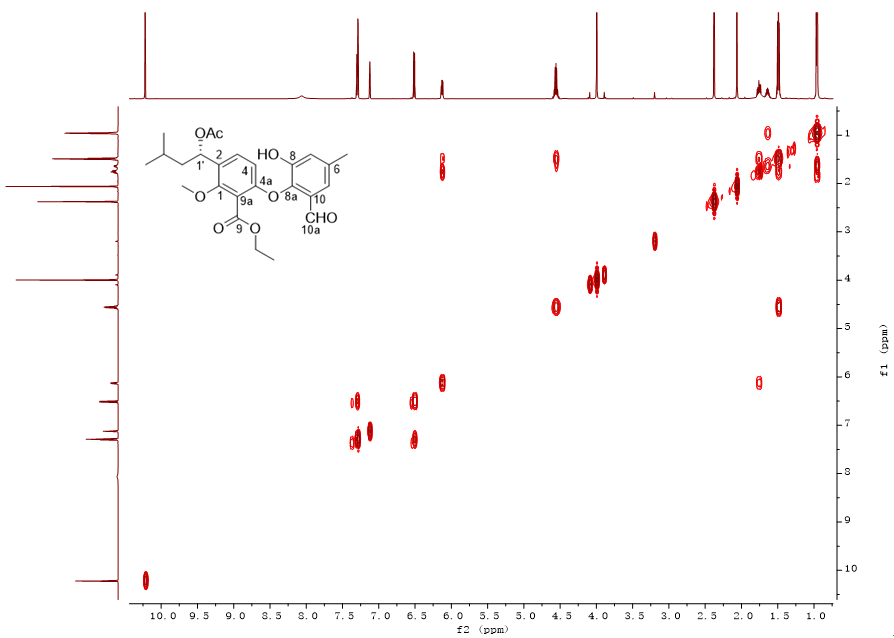


**Fig. S44** ^1^H-^1^H COSY (600 MHz) spectrum of **5** in CDCl_3_.


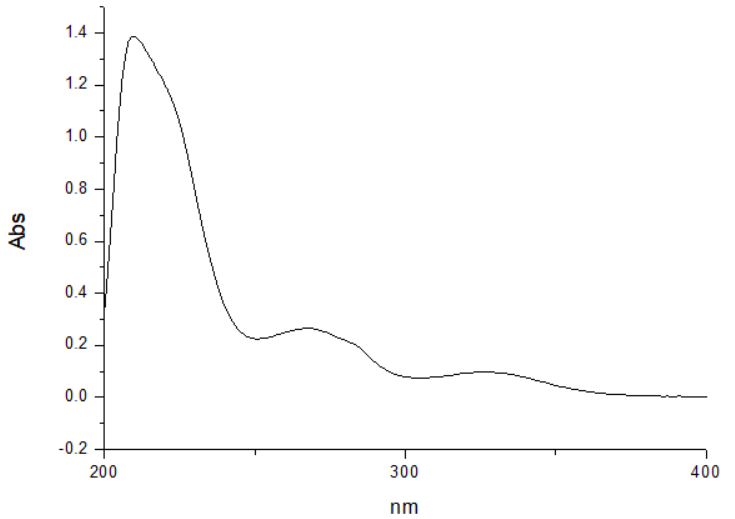


**Fig. S45** UV spectrum of **5** in MeOH.


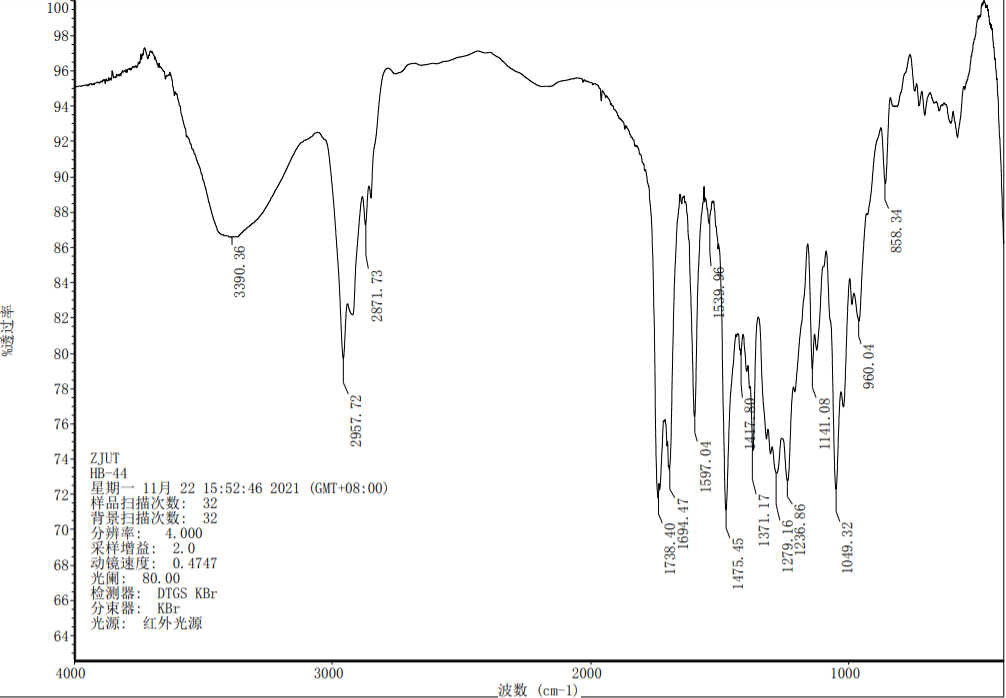


**Fig. S46** IR spectrum of **5**.


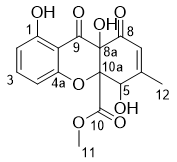


**Fig. S47** HRESIMS spectrum of **6**.


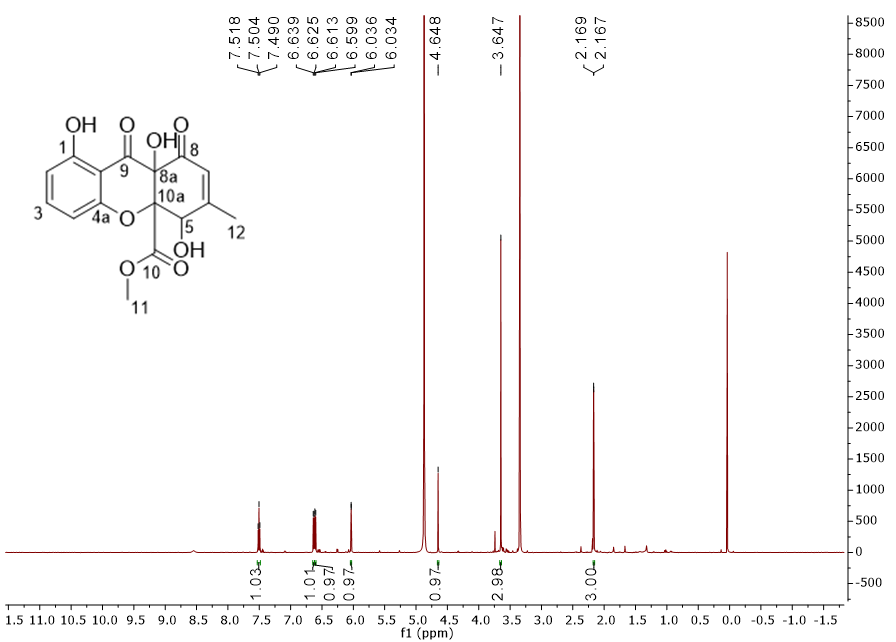


**Fig. S48** ^1^H NMR (600 MHz) spectrum of **6** in MeOH-*d_4_*.


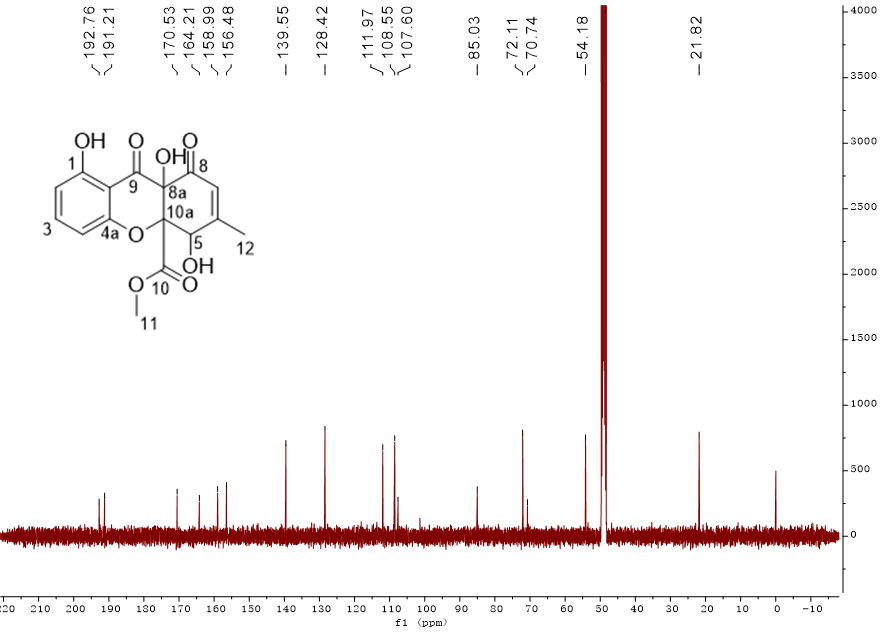


**Fig. S49** ^13^C NMR (150 MHz) spectrum of **6** in MeOH-*d_4_*.


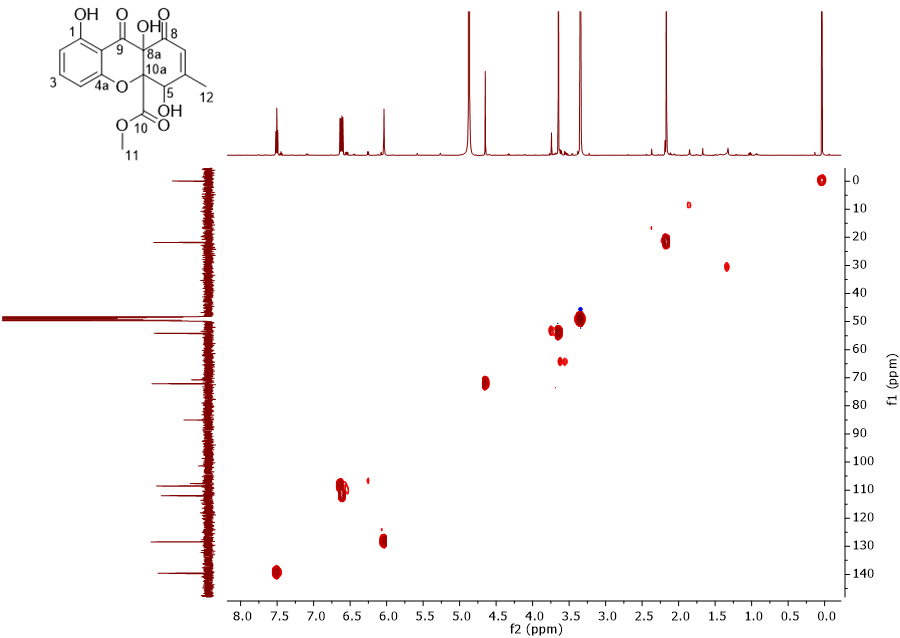


**Fig. S50** HSQC (600 MHz) spectrum of **6** in MeOH-*d_4_*.


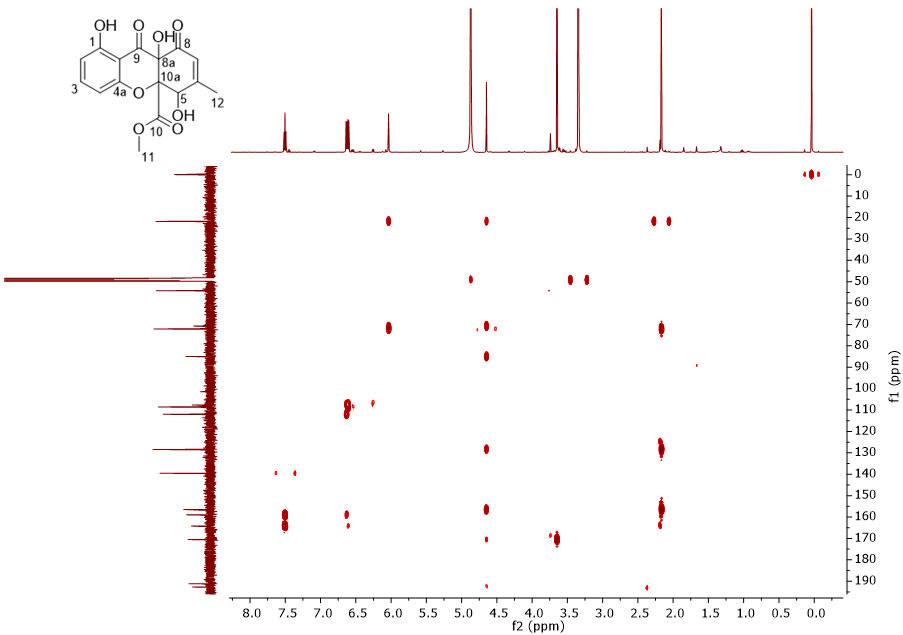


**Fig. S51** HMBC (600 MHz) spectrum of **6** in MeOH-*d_4_*.


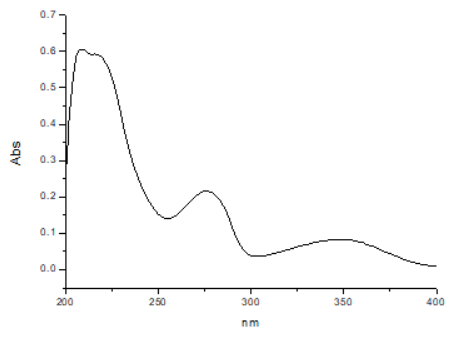


**Fig. S52** UV spectrum of **6** in MeOH.


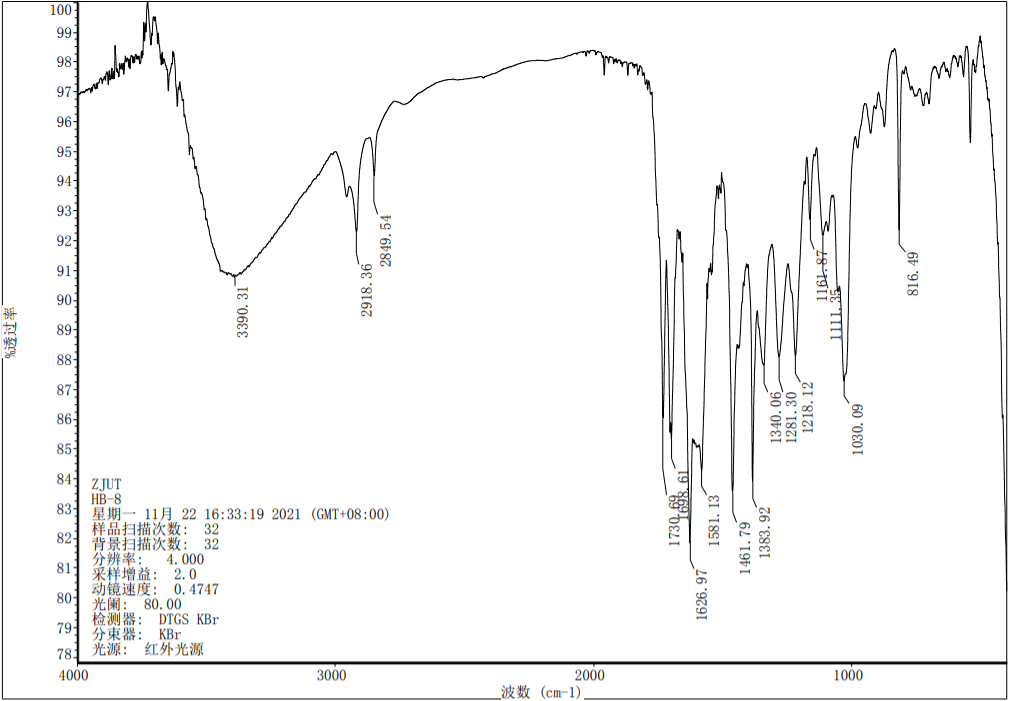


**Fig. S53** IR spectrum of **6**.


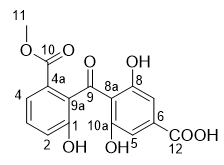


**Fig. S54** HRESIMS spectrum of **7**.


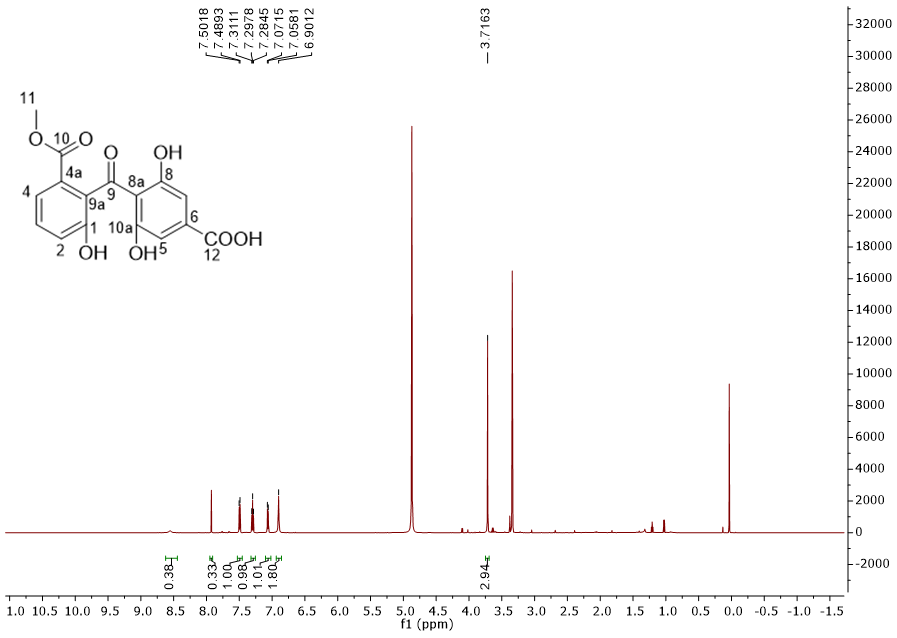


**Fig. S55** ^1^H NMR (600 MHz) spectrum of **7** in MeOH-*d_4_*.


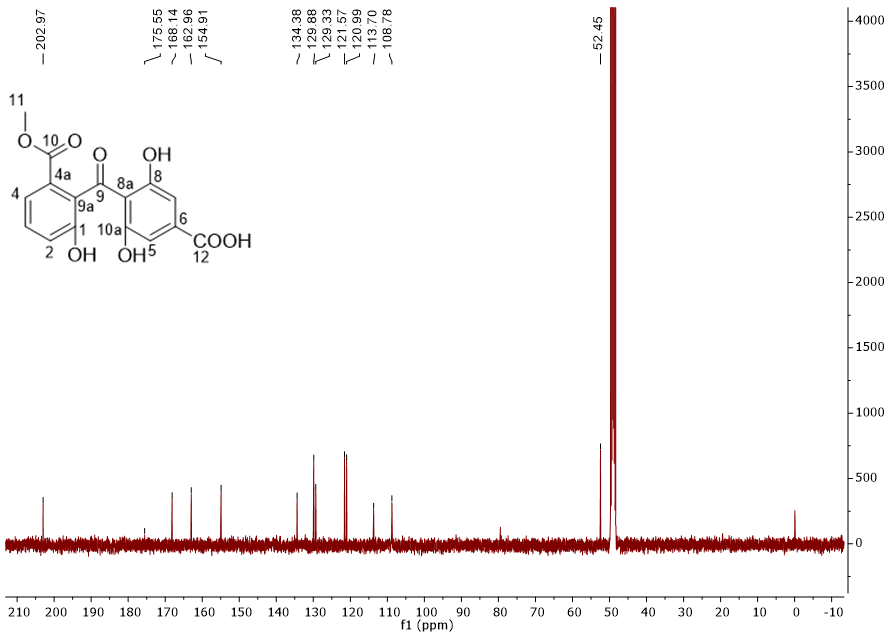


**Fig. S56** ^13^C NMR (150 MHz) spectrum of **7** in MeOH-*d_4_*.


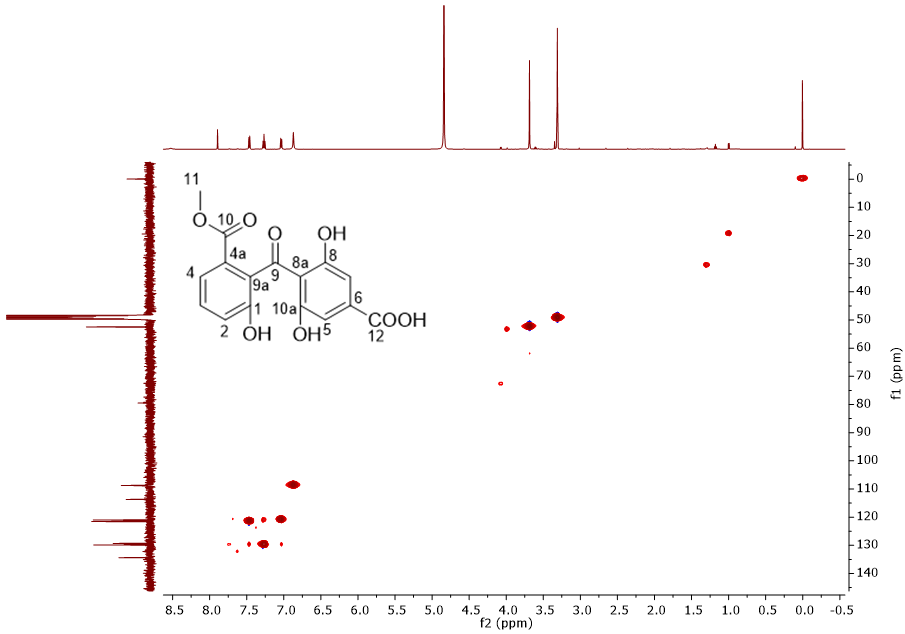


**Fig. S57** HSQC (600 MHz) spectrum of **7** in MeOH-*d_4_*.


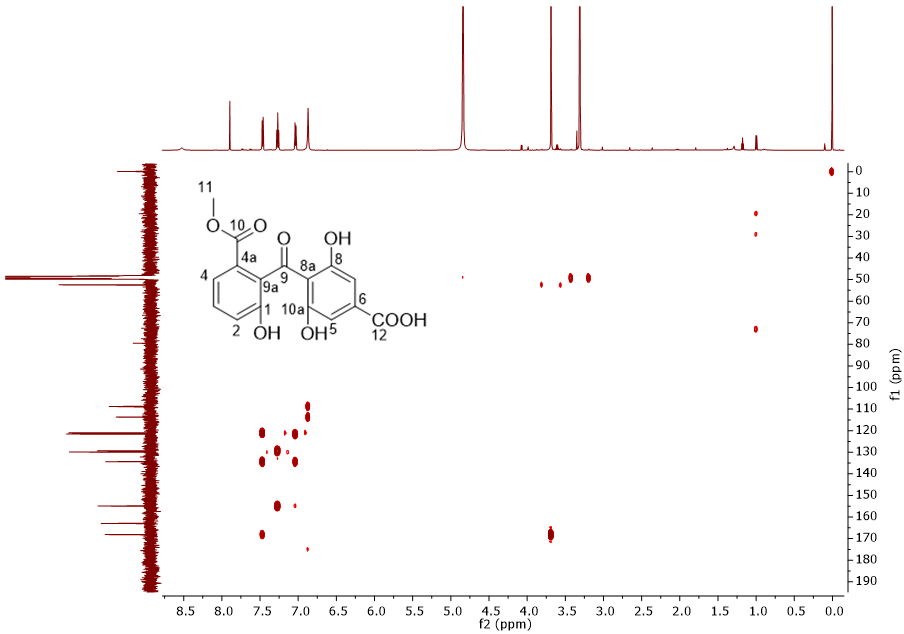


**Fig. S58** HMBC (600 MHz) spectrum of **7** in MeOH-*d_4_*.


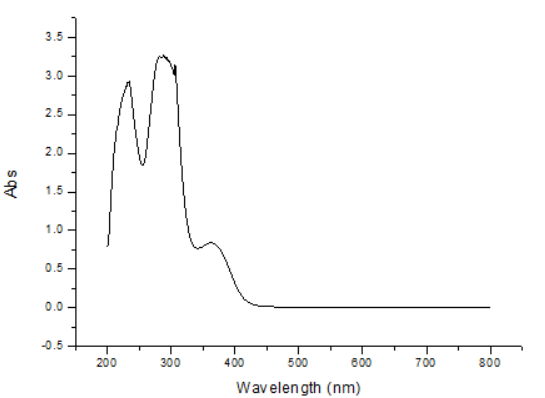


**Fig. S59** UV spectrum of **7** in MeOH.


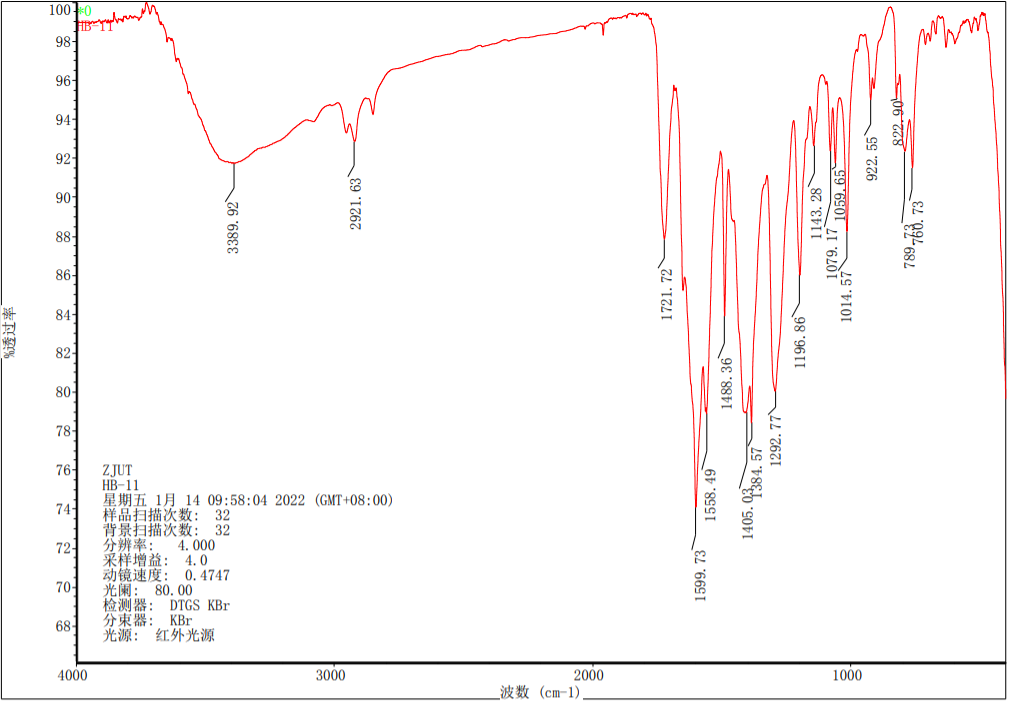


**Fig. S60** IR spectrum of **7**.


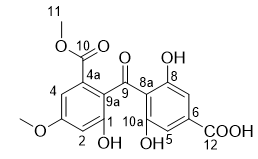


**Fig. S61** HRESIMS spectrum of **8**.


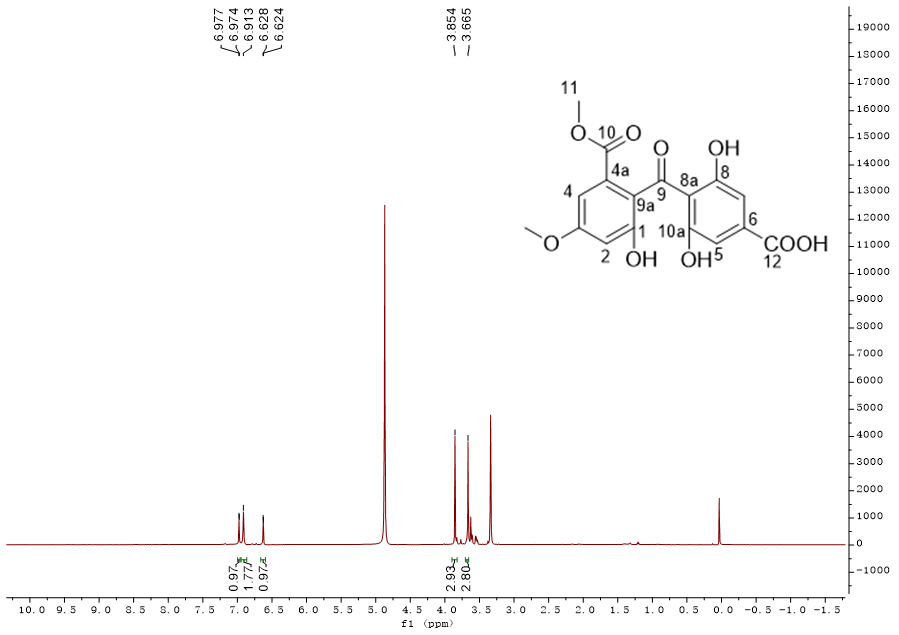


**Fig. S62** ^1^H NMR (600 MHz) spectrum of **8** in MeOH-*d_4_*.


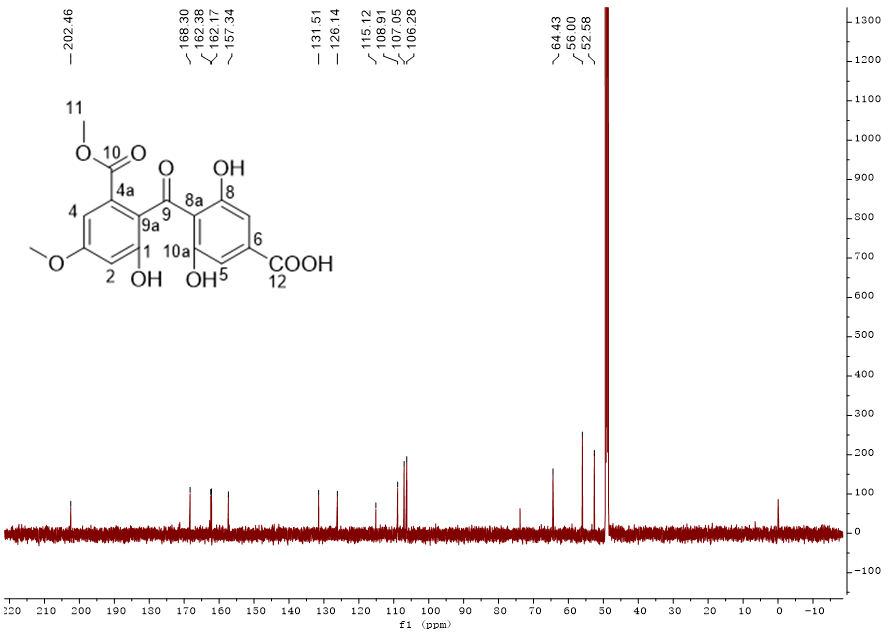


**Fig. S63** ^13^C NMR (150 MHz) spectrum of **8** in MeOH-*d_4_*.


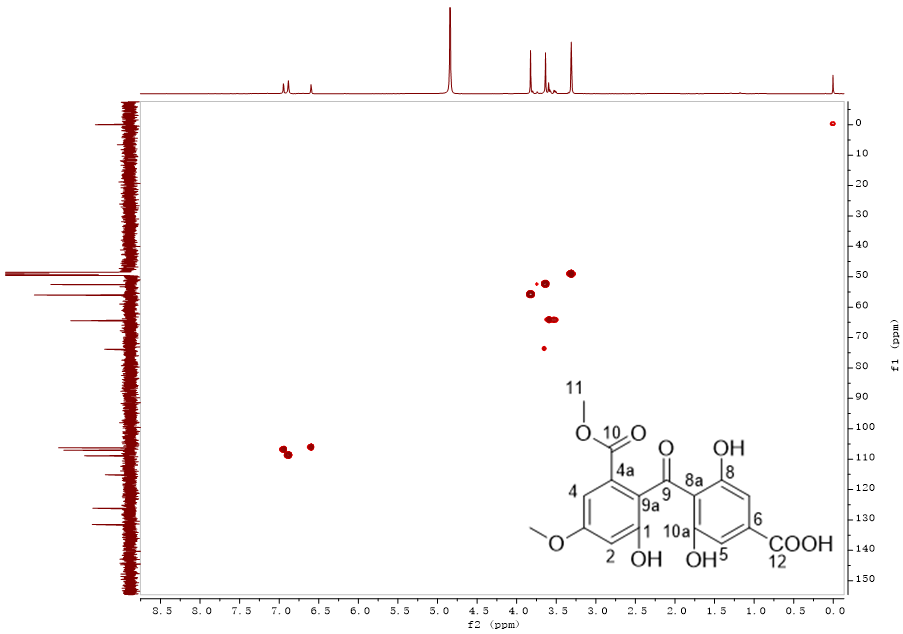


**Fig. S64** HSQC (600 MHz) spectrum of **8** in MeOH-*d_4_*.


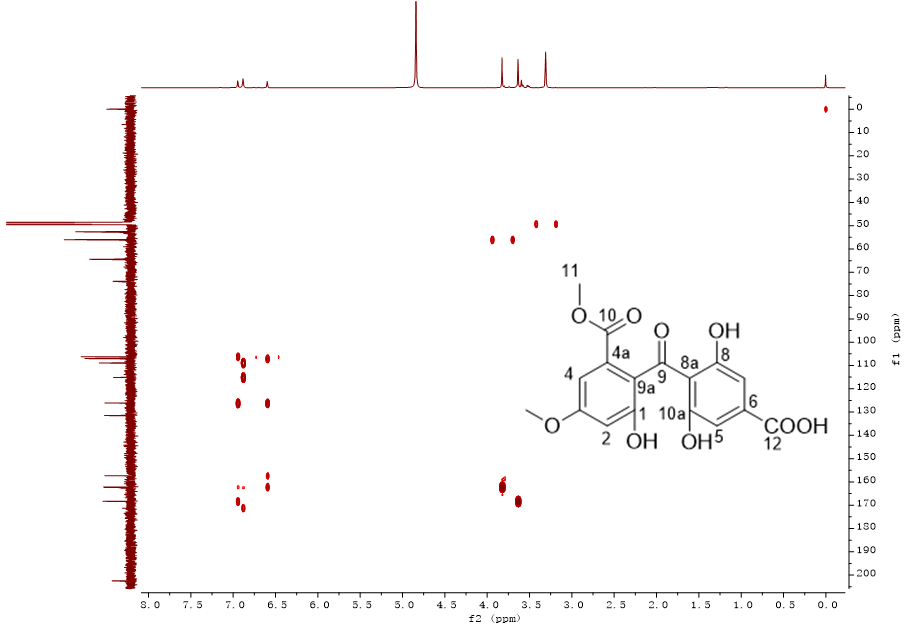


**Fig. S65** HMBC (600 MHz) spectrum of **8** in MeOH-*d_4_*.


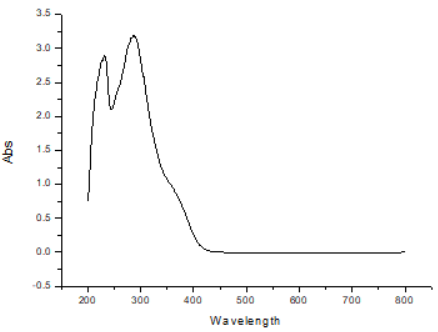


**Fig. S66** UV spectrum of **8** in MeOH.


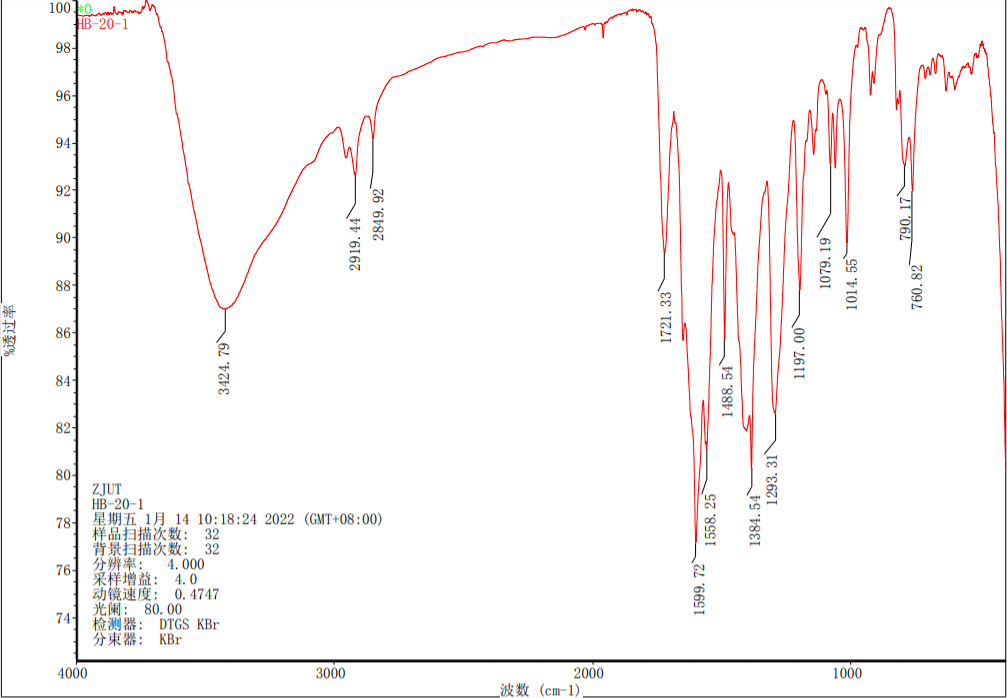


**Fig. S67** IR spectrum of **8**.

**
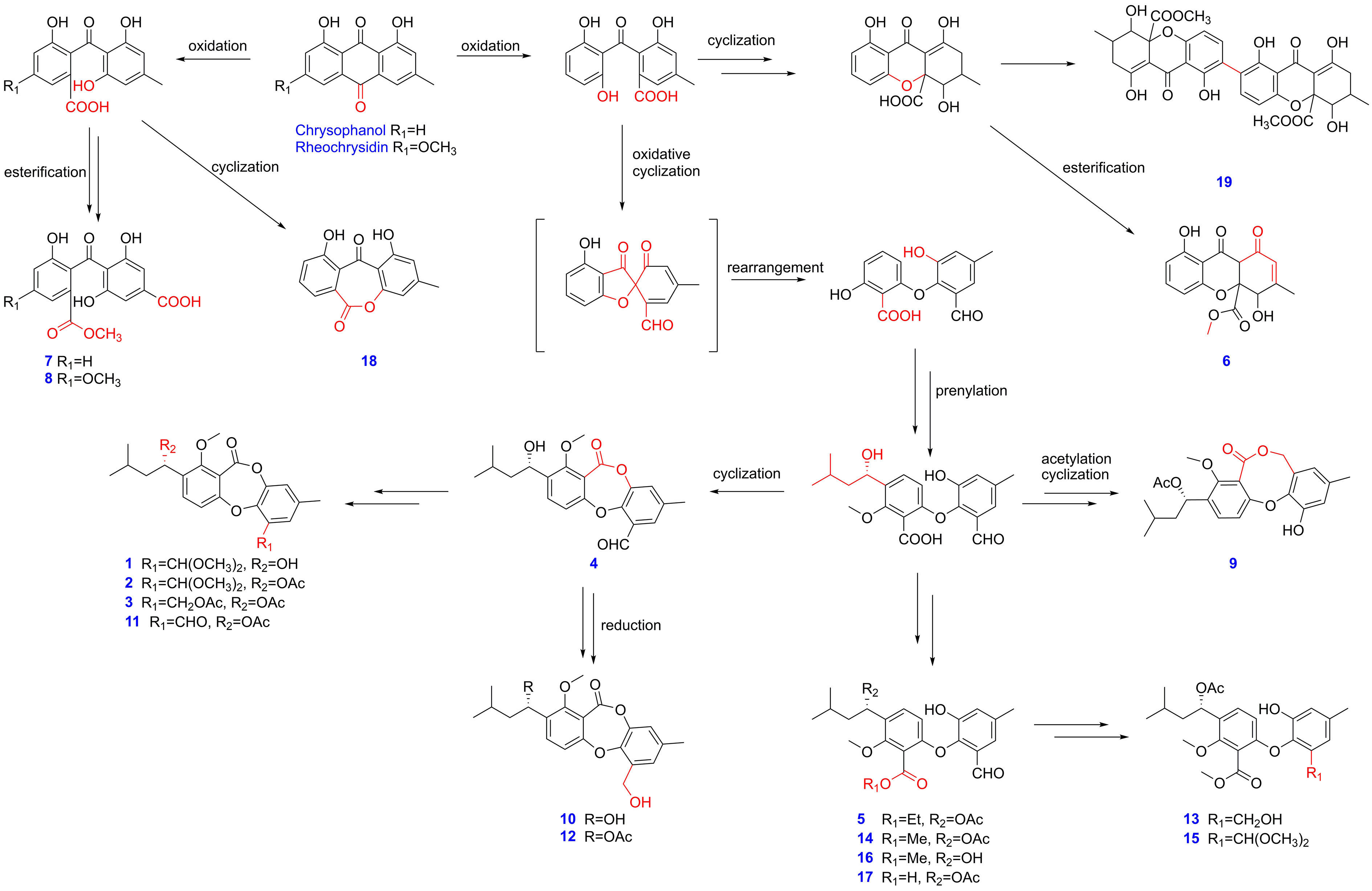
**

**Fig. S68** The proposed biogenetic relationship of the isolated metabolites

The experimental procedure of alkaline hydrolysis and Mosher’ method

Compound **2** (3 mg) was dissolved in methanol (2 mL) and LiOH (1.5 mg) was added to the solution. The solution was allowed to stand at room temperature for 4 h. Then, the solution was filtered and the filtrate was evaporated to yield alkaline hydrolysis product (**2a**). **2a** was dissolved in pyridine (0.4 mL) and the solution was divided into two equal portions. Each portion was added with (*R*)- and (*S*)-MTPA-Cl (3 μL), respectively. After 12 h, the solvent was evaporated to yield (*S*)- and (*R*)-MTPA ethers.

Antimicrobial Assays

The antimicrobial assay and the determination of the MIC values were performed following broth the microdilution method in 96-well plates. The specific experiment was as follows: the initial cultures for *H. pylori* strains were maintained on Brain Heart Infusion (BHI) medium and other pathogenic strains were maintained on Luria Broth (LB) agar. A single bacterial colony was picked and suspended in LB/BHI broth to approximately 10^6^ CFU/mL. Each compound was dissolved in DMSO and serially diluted to 9 different concentrations (16.0, 8.0, 4.0, 2.0, 1.0, 0.5, 0.25, 0.125, 0.0625 μg/mL) using LB broth on a 96-well plate. An aliquot (10 μL) of bacterial suspension was then added to each well. Sterile water was used as the negative control. Amoxicillin, vancomycin, and amphotericin B were used as the positive controls for *H. pylori*, *S.*, *aureus*, *C.*, and *albicans* strains, respectively. Ampicillin was used as the positive control for the other strains. The plate was incubated at 37°C aerobically for 24 h. The next day, growth of the test organisms was visually observed, and the MIC values were determined at the lowest concentration of compound where no visible growth of test organisms. The results were uniformly converted to μM.

Table S1 The inhibitory activity against Gram-negative bacteria of compounds 1-24

| *Compound* | *H. pylori* 26695 | *H. pylori* G27 | *H. pylori* 159 | *H. pylori* 129 | *E. coli* MG1655 | *P. aeruginos*a PAO1 | *A. baumannii* ATCC19606 | *K. neumoniae* ATCC35657 | *S. typhimurium 14028s* | *S. dysenteriae* |
| --- | --- | --- | --- | --- | --- | --- | --- | --- | --- | --- |
| **1** | >38.46 | >38.46 | >38.46 | >38.46 | >38.46 | >38.46 | >38.46 | >38.46 | >38.46 | >38.46 |
| **2** | >34.93 | >34.93 | >34.93 | >34.93 | >34.93 | >34.93 | >34.93 | >34.93 | >34.93 | >34.93 |
| **3** | >35.09 | >35.09 | >35.09 | >35.09 | >35.09 | >35.09 | >35.09 | >35.09 | >35.09 | >35.09 |
| **4** | >43.24 | >43.24 | >43.24 | >43.24 | >43.24 | >43.24 | >43.24 | >43.24 | >43.24 | >43.24 |
| **5** | **34.93** | **34.93** | >34.93 | >34.93 | >34.93 | >34.93 | >34.93 | >34.93 | >34.93 | >34.93 |
| **6** | >47.90 | >47.90 | >47.90 | >47.90 | >47.90 | >47.90 | >47.90 | >47.90 | >47.90 | >47.90 |
| **7** | >48.19 | >48.19 | >48.19 | >48.19 | >48.19 | >48.19 | >48.19 | >48.19 | >48.19 | >48.19 |
| **8** | >44.20 | >44.20 | >44.20 | >44.20 | >44.20 | >44.20 | >44.20 | >44.20 | >44.20 | >44.20 |
| **9** | >38.65 | **4.83** | >38.65 | **2.41** | >38.65 | >38.65 | >38.65 | >38.65 | >38.65 | >38.65 |
| **10** | **10.75** | **10.75** | **5.38** | **5.38** | >43.01 | >43.01 | >43.01 | >43.01 | >43.01 | >43.01 |
| **11** | >38.83 | >38.83 | >38.83 | >38.83 | >38.83 | >38.83 | >38.83 | >38.83 | >38.83 | >38.83 |
| **13** | >35.87 | >35.87 | >35.87 | >35.87 | >35.87 | >35.87 | >35.87 | >35.87 | >35.87 | >35.87 |
| **14** | **36.04** | **36.04** | >36.04 | >36.04 | >36.04 | >36.04 | >36.04 | >36.04 | >36.04 | >36.04 |
| **15** | >32.65 | >32.65 | >32.65 | >32.65 | >32.65 | >32.65 | >32.65 | >32.65 | >32.65 | >32.65 |
| **16** | >39.80 | >39.80 | >39.80 | >39.80 | >39.80 | >39.80 | >39.80 | >39.80 | >39.80 | >39.80 |
| **17** | >37.21 | >37.21 | >37.21 | >37.21 | >37.21 | >37.21 | >37.21 | >37.21 | >37.21 | >37.21 |
| **18** | >59.26 | >59.26 | >59.26 | >59.26 | >59.26 | >59.26 | >59.26 | >59.26 | >59.26 | >59.26 |
| **19** | **0.20** | **0.20** | **1.57** | **1.57** | >25.08 | >25.08 | >25.08 | >25.08 | >25.08 | >25.08 |
| **20** | >32.65 | >32.65 | >32.65 | >32.65 | >32.65 | >32.65 | >32.65 | >32.65 | >32.65 | >32.65 |
| **21** | >114.3 | >114.3 | >114.3 | >114.3 | >114.3 | >114.3 | >114.3 | >114.3 | >114.3 | >114.3 |
| **22** | >115.9 | >115.9 | >115.9 | >115.9 | >115.9 | >115.9 | >115.9 | >115.9 | >115.9 | >115.9 |
| **23** | >76.19 | >76.19 | >76.19 | >76.19 | >76.19 | >76.19 | >76.19 | >76.19 | >76.19 | >76.19 |
| **24** | >77.67 | >77.67 | >77.67 | >77.67 | >77.67 | >77.67 | >77.67 | >77.67 | >77.67 | >77.67 |
| Positive Control | 0.14^a^ | 1.19^a^ | 38.14^a^ | 0.30^a^ | 11.45^d^ | >45.79^d^ | >183.2^d^ | >45.79^d^ | 2.86^d^ | >45.79^d^ |

^a,b,c,d^ The concentrate of Amoxicillin, Vancomycin, Amphotericin B and Ampicillin, respectively.

Table S2 The inhibitory activity against Gram-positive bacteria, Mycobacterium and Fungus of compounds 1-24

|  | Gram-positive bacteria | | | | | | |  | Mycobacterium |  | Fungus | |
| --- | --- | --- | --- | --- | --- | --- | --- | --- | --- | --- | --- | --- |
| *Compound* | *E. faecalis FA2-2* | *E. faecium ATCC19434* | *B. subtilis 168* | *S. aureus ATCC25923* | *S. aureus NEWMAN* | *S. aureus USA300* | *S. aureus NRS 271* |  | *M. smegmatis* ATCC 607 |  | *C. albicans ATCC SC5314* | *C. albicans clinical isolates YY-1-4* |
| **1** | >38.46 | >38.46 | >38.46 | >38.46 | >38.46 | >38.46 | >38.46 |  | >38.46 |  | >38.46 | >38.46 |
| **2** | >34.93 | >34.93 | >34.93 | >34.93 | >34.93 | >34.93 | >34.93 |  | >34.93 |  | >34.93 | >34.93 |
| **3** | >35.09 | >35.09 | >35.09 | >35.09 | >35.09 | >35.09 | >35.09 |  | >35.09 |  | >35.09 | >35.09 |
| **4** | >43.24 | >43.24 | >43.24 | >43.24 | >43.24 | >43.24 | >43.24 |  | >43.24 |  | >43.24 | >43.24 |
| **5** | >34.93 | >34.93 | >34.93 | >34.93 | >34.93 | >34.93 | >34.93 |  | >34.93 |  | >34.93 | >34.93 |
| **6** | >47.90 | >47.90 | >47.90 | >47.90 | >47.90 | >47.90 | >47.90 |  | >47.90 |  | >47.90 | >47.90 |
| **7** | >48.19 | >48.19 | >48.19 | >48.19 | >48.19 | >48.19 | >48.19 |  | >48.19 |  | >48.19 | >48.19 |
| **8** | >44.20 | >44.20 | >44.20 | >44.20 | >44.20 | >44.20 | >44.20 |  | >44.20 |  | >44.20 | >44.20 |
| **9** | >38.65 | >38.65 | >38.65 | >38.65 | >38.65 | >38.65 | >38.65 |  | >38.65 |  | >38.65 | >38.65 |
| **10** | >43.01 | >43.01 | >43.01 | >43.01 | >43.01 | >43.01 | >43.01 |  | >43.01 |  | >43.01 | >43.01 |
| **11** | >38.83 | >38.83 | >38.83 | >38.83 | **38.83** | >38.83 | >38.83 |  | >38.83 |  | >38.83 | >38.83 |
| **13** | >35.87 | >35.87 | >35.87 | >35.87 | >35.87 | >35.87 | >35.87 |  | >35.87 |  | >35.87 | >35.87 |
| **14** | >36.04 | >36.04 | >36.04 | >36.04 | >36.04 | >36.04 | >36.04 |  | >36.04 |  | >36.04 | >36.04 |
| **15** | >32.65 | >32.65 | >32.65 | >32.65 | >32.65 | >32.65 | >32.65 |  | >32.65 |  | >32.65 | >32.65 |
| **16** | >39.80 | >39.80 | >39.80 | >39.80 | >39.80 | >39.80 | >39.80 |  | >39.80 |  | >39.80 | >39.80 |
| **17** | >37.21 | >37.21 | >37.21 | >37.21 | >37.21 | >37.21 | >37.21 |  | >37.21 |  | >37.21 | >37.21 |
| **18** | >59.26 | >59.26 | >59.26 | >59.26 | >59.26 | >59.26 | >59.26 |  | >59.26 |  | >59.26 | >59.26 |
| **19** | >25.08 | >25.08 | >25.08 | >25.08 | >25.08 | >25.08 | >25.08 |  | >25.08 |  | >25.08 | >25.08 |
| **20** | >32.65 | >32.65 | >32.65 | >32.65 | >32.65 | >32.65 | >32.65 |  | >32.65 |  | >32.65 | >32.65 |
| **21** | >114.3 | >114.3 | >114.3 | >114.3 | >114.3 | >114.3 | >114.3 |  | >114.3 |  | >114.3 | >114.3 |
| **22** | >115.9 | >115.9 | >115.9 | >115.9 | >115.9 | >115.9 | >115.9 |  | >115.9 |  | >115.9 | >115.9 |
| **23** | >76.19 | >76.19 | >76.19 | >76.19 | >76.19 | >76.19 | >76.19 |  | >76.19 |  | >76.19 | >76.19 |
| **24** | >77.67 | >77.67 | >77.67 | >77.67 | >77.67 | >77.67 | >77.67 |  | >77.67 |  | >77.67 | >77.67 |
| Positive Control | 2.86^d^ | 2.86^d^ | 0.17^d^ | 0.69^b^ | 1.38^b^ | 0.69^b^ | 0.69^b^ |  | 183.2^d^ |  | 2.16^c^ | 2.16^c^ |

^a,b,c,d^ The concentrate of Amoxicillin, Vancomycin, Amphotericin B and Ampicillin, respectively.

Cytotoxicity Assay

Compounds were tested for cytotoxicity against the A-549 human lung cancer, the Bel -7402 human liver cancer, and the HCT-116 human colon cancer cell lines using the SRB assay. All the cancer cell lines were purchased from American Type Culture Collection (ATCC) and cultured in the ATCC-specified medium supplemented with 10% heat-inactivated phosphate-buffered saline (PBS), penicillin (100 units/mL), and streptomycin (100 μg/mL) in a humidified atmosphere containing 5% CO_2_ at 37°C. Logarithmic growth phase cells were selected and digested by trypsinase to adjust the cell concentration to 2×10^4^ cells/mL in RPMI 1640 medium containing 10% fetal bovine serum. The cells were inoculated in a 96-well culture plate with 190 μL cell suspension per well at 37°C and 5% CO_2_ for 24 h. Then, 10 μL sample solution was added to the drug treatment well (final concentration: compound 5.0 μg/mL) and the positive drug well was treated with 5-fluorouracil (purity: 99%, National Institutes for Food and Drug Control) with the final concentration of 5.0 μg/mL; the control well was treated with medium containing an equal volume of solvent. The 96-well culture plate was cultured at 37°C with 5% CO_2_ for 3 days. The medium was then discarded and 100 μL 50% pre-processed TCA was gently added at 4°C to fix cells for 5 min, before moving to 4°C for 1 h. The fixative was poured out, and the plate wells washed with distilled water 5 times to remove TCA, followed by air drying for 1 h before adding 80 μL 0.4% SRB solution to each well and staining for 30 min at room temperature prior to discarding the dye and washing the unbound SRB with 1% acetic acid 5 times prior to air drying. 150 μL 10 mmol/L Trisbase (pH 10.5) was then added to dissolve on the micro oscillator for 5 min and measured at 510 nm by M5 microplate analyzer. The inhibitory rates of cell proliferation (%) were calculated as [1-(A_treated_/A_control_)]×100%. Three independent repeated trials were conducted for each compound (*n* = 3). IC_50_ values were determined with the Logit method from the results of six concentrations of each compound.

Table S3 The initial screening inhibition rate of the tested compounds

| Compounds* | Inhibition ratio (%) | | |
| --- | --- | --- | --- |
|  | A549 | Bel-7402 | HCT-116 |
| **1** | 19.44 | -5.25 | -2.93 |
| **2** | 15.43 | -5.25 | 3.59 |
| **3** | 25.28 | -5.73 | -5.75 |
| **5** | 23.26 | -3.97 | 3.17 |
| **6** | 23.84 | -7.58 | -2.78 |
| **10** | 2.69 | 1.67 | 11.68 |
| **11** | 7.15 | 13.56 | 27.33 |
| **13** | 19.33 | -4.04 | 12.84 |
| **14** | -12.18 | 2.42 | 1.37 |
| **15** | 17.35 | -4.01 | 6.38 |
| **19** | 27.05 | 73.48 | 87.64 |
| **20** | 22.98 | -0.10 | 1.87 |
| **23** | 20.89 | -4.30 | 13.00 |
| **24** | 20.53 | -3.03 | 4.55 |
| **5-FU** | 90.42 | 56.41 | 82.76 |
| *Test concentration: 5.0 μg/mL | | | |
